# Distinct prosocial behaviors associated with persistent symptom trajectories in psychiatric disorders

**DOI:** 10.1038/s41398-026-04515-3

**Published:** 2026-10-05

**Authors:** Simon Jangard, Joana B. Vieira, Maria Å. Garke, Olivia Ojala, Kristoffer N. T. Månsson, Maria Hedman-Lagerlöf, Karolina Sörman, Samir El Alaoui, Alexander Rozental, David Forsström, Johan Lundin, Shervin Shahnavaz, Tobias Lundgren, Andreas Olsson, Nitya Jayaram-Lindström

**Affiliations:** 1 Centre for Psychiatry Research, Department of Clinical Neuroscience, Karolinska Institutet & Stockholm Health Care Services, Stockholm County Council, Stockholm, Sweden; 2 Division of Psychology, Department of Clinical Neuroscience, Karolinska Institutet, Stockholm, Sweden; 3 Division of Experimental Psychology, Faculty of Social and Behavioral Sciences, Helmholtz Institute, Utrecht University, Utrecht, Netherlands; 4 Department of Clinical Psychology and Psychotherapy, Babeș-Bolyai University, Cluj-Napoca, Romania; 5 Department of Health, Education, and Technology, Luleå University of Technology, Luleå, Sweden

**Keywords:** Human behaviour, Psychiatric disorders

## Abstract

Prosocial behavior, the tendency to act for others’ benefit, is essential for mental health. Although social contexts strongly shape prosocial behavior in healthy individuals, its relevance across psychiatric disorders remains unclear. We examined whether context-dependent prosocial behaviors were associated with baseline psychiatric symptoms and longitudinal symptom trajectories. In a one-year longitudinal cohort study, Swedish adults (*n* = 4484) with at least one self-reported psychiatric disorder completed standardized questionnaires of six prosocial behavior subtypes at baseline, followed by monthly reports of alcohol use, substance use, anxiety, and depressive symptoms. Latent class linear mixed models identified longitudinal symptom trajectories, and logistic regression models investigated their associations with baseline prosocial behavior subtypes. All models adjusted for demographic covariates and shared variance across psychiatric symptom domains. Three prosocial behavior subtypes showed longitudinal associations with stable psychiatric symptom trajectory classes. Higher anonymous prosociality was associated with lower odds of belonging to the stable high alcohol use trajectory (OR = 0.89, *p* = 0.013). Higher public prosociality was associated with higher odds of belonging to the stable high substance use trajectory (OR = 1.15, *p* = 0.036). Higher emotional prosociality was associated with higher odds of belonging to the stable high anxiety trajectory (OR = 1.25, *p* < 0.001). No prosocial subtype was associated with depressive symptom trajectories. Overall, context-dependent prosocial behaviors were associated with persistent levels of alcohol use, substance use, and anxiety symptoms. These findings suggest that specific prosocial behaviors may refine transdiagnostic models of social dysfunction by indexing stable patterns of psychiatric symptom burden.

## Introduction

Difficulties in social functioning are common across psychiatric disorders and associated with symptom severity and lower quality of life [1–5]. Specific social behaviors intended to help or benefit others (i.e., prosocial behaviors) play an important role in maintaining cooperation [6], interpersonal relationships [7], and psychological well-being [8]. Importantly, prosocial behavior relies on neurocognitive processes that are frequently implicated in psychiatric disorders, including reward valuation, social cognition, empathy, and affective processing [9]. Consequently, disruptions in these systems may contribute to both psychiatric symptoms and altered prosocial behavior. In turn, reduced engagement in prosocial behaviors may impair social connectedness and access to supportive social relationships, potentially contributing to the maintenance of psychiatric symptoms. Yet, the associations between prosocial behavior and psychiatric symptoms over time remain poorly understood. Clarifying these associations may help identify mechanisms of risk and resilience that operate across disorders and inform behavioral and preventive mental health interventions.

Research on prosocial behavior in psychiatric disorders has mainly been cross-sectional and focused on altruism, a subtype of prosociality defined as helping others without any personal benefit or even at a personal cost [10]. For example, studies show that individuals with alcohol use disorder (AUD) [11] and cocaine use disorder [12] demonstrate reduced altruism in comparison to healthy individuals. However, a longitudinal community sample followed from childhood to young adulthood found no prospective association between early altruism and later substance use [13], suggesting that reduced altruism observed in SUD populations may not necessarily reflect a premorbid risk factor. However, differences in study populations and outcomes (substance use versus SUD) complicate direct comparisons. Next, cross-sectional studies suggest reduced altruistic behavior among individuals with major depressive disorder (MDD) relative to healthy controls [14, 15]. In contrast, increased altruism has also been associated with the later onset of MDD [16]. Evidence from healthy cohorts linking anxiety to altruism is likewise inconsistent: some studies show anxiety symptoms to be associated with lower levels of altruism [17, 18], whereas others associated acute anxiety in responses to critical events with higher altruism [15]. Finally, research on prosocial behavior has also been examined in personality disorders [19], although studies have typically focused on broader measures of prosociality rather than altruism specifically. In particular, research examining agreeableness from the five-factor model suggests that antisocial and borderline personality disorders are associated with lower levels of prosocial behavior, which may contribute to the interpersonal difficulties that characterize these disorders [20]. Collectively, these findings suggest that prosocial behavior covaries with psychiatric symptoms. However, previous studies have largely investigated single psychiatric symptom domains in isolation, and longitudinal evidence has been limited to healthy cohorts.

Converging findings from behavioral and neuroimaging research indicate that prosocial behavior is not a uniform construct, but rather multifaceted in nature and comprising partly dissociable motivational processes and neural systems [11, 21–23]. For example, empathy-driven helping, norm compliance, and strategic cooperation recruit partially dissociable neural circuits within the prefrontal cortex, striatum, and temporoparietal regions. Importantly, prosocial behavior is highly context-dependent [21, 24, 25]. A useful behavioral framework in this regard is the Prosocial Tendencies Model [25], which distinguishes six subtypes of prosociality that differ across social and motivational contexts: altruistic, anonymous, compliant, dire, emotional, and public (see Methods and Materials for full definitions). In non-clinical samples, public prosocial behavior (i.e., helping in front of others) is associated with higher substance use [26], anxiety [17, 27], and depressive symptoms [27], suggesting the relevance of social behaviors strongly dependent on the approval of others for understanding psychiatric symptoms. Moreover, emotional prosocial behavior (i.e., helping when affectively aroused) is associated with symptoms of anxiety in healthy individuals [15, 17, 27]. Taken together, these observations suggest that context-dependent forms of prosocial behavior may show differential associations with psychiatric symptom variation and may help identify social processes relevant to the development and maintenance of psychiatric disorders.

To elucidate how prosocial behavior relates to heterogeneity in psychiatric symptoms, the present study examined the associations between six prosocial subtypes and 1-year trajectories of alcohol use, other substance use, anxiety, and depressive symptoms in a large clinical sample of adults with psychiatric disorders (N = 4484). Given the heterogeneity and partial inconsistency of the existing literature, we formulated a priori hypotheses only for prosocial subtypes with relatively convergent prior evidence, while treating remaining associations as exploratory. Based on cross-sectional and longitudinal evidence from psychiatric cohorts and non-clinical samples, we hypothesized that (1) lower altruistic prosociality at baseline would predict higher alcohol [11, 26, 28], substance use [12, 13, 26, 28], anxiety [16, 17, 27], and depressive symptoms [27, 29], reflecting reduced concern for others and diminished social engagement; (2) higher emotional prosocial behavior would predict anxiety [17, 27] and depressive symptoms [17, 27], given its association with heightened emotional responsivity; and (3) higher public prosocial behavior (i.e., helping in front of others) would predict substance use [26], anxiety [17, 27], and depressive symptoms [17, 27], reflecting a greater dependence on social approval and external validation. Finally, given the lack of consistent evidence, we did not formulate hypotheses regarding associations between the remaining prosocial subtypes (anonymous, compliant, and dire helping) and subsequent substance use or psychiatric symptoms.

## Methods and materials

### Study design and participants

This prospective longitudinal one-year cohort study enrolled adults in Sweden with a current or past psychiatric disorder. Participants (n = 4484; 71.1% female; mean age = 35.8 [SD = 12.3], Table 1) were assessed monthly via the REDCap online platform at Karolinska Institutet [30]. Recruitment occurred through outpatient psychiatric clinics and online advertisements between July 2020 and June 2021, with follow-up completed in June 2022. Attrition at one-year follow-up was 69.4% (i.e., 1376 participants remained, see Table 2). Detailed recruitment procedures and COVID-19 control measures are provided in Supplementary Methods.

**Table 1:** Demographics and Clinical Characteristics at Baseline (*n* = 4484^a^).

Characteristics	
Gender, No. (%)	
Female	3189 (71.1)
Male	1065 (23.8)
Other	230 (5.1)
Socioeconomic Status, Education, No. (%)	
Elementary school	309 (6.9)
High school	1745 (38.9)
Undergraduate Degree or Higher	2430 (54.2)
Prosocial Behavior (PTM), Mean (SD)	
Altruistic Prosocial Behavior	23.1 (2.5)
Anonymous Prosocial Behavior	13.0 (4.7)
Compliant Prosocial Behavior	7.4 (2.0)
Dire Prosocial Behavior	10.4 (2.9)
Emotional Prosocial Behavior	11.7 (3.5)
Public Prosocial Behavior	5.7 (2.4)
**Psychiatric Attributes**	
Alcohol Use (AUDIT), Mean (SD)	7.9 (5.9)
Substance Use (DUDIT), Mean (SD)	1.5 (4.4)
Anxiety Symptoms (GAD-7), Mean (SD)	12.0 (5.7)
Depressive Symptoms (PHQ-9), Mean (SD)	15.3 (6.7)
Lifetime diagnosis of alcohol or substance use disorder, No. (%)	414 (9.2)
Lifetime diagnosis of anxiety disorder, No. (%)	3199 (71.3)
Lifetime diagnosis of depressive disorder, No. (%)	3517 (78.4)
Current psychiatric treatment No. (%)	
Psychotherapy/Counselling	2242 (50.0)
Pharmacotherapy	1090 (24.3)

*SD*, Standard Deviation; *PTM*, Prosocial Tendencies Measure; *AUDIT*, Alcohol Use Disorder Identification Test; *DUDIT*, Drug Use Disorder Identification Test; *GAD-7*, Generalized Anxiety Disorder-7; *PHQ-9*, Patient Health Questionnaire-9.

^a^Included participants in this study were required to complete the full baseline assessment, reducing the total number of participants reported in the main study [32].

**Table 2:** Psychiatric Symptom Load at Baseline and Follow-up.

Month	B	1	2	3	4	5	6	7	8	9	10	11	12
Total No.	4484	2793	2494	2285	2116	2007	1846	1710	1673	1579	1481	1421	1376
Alcohol Use^a^, %													
None	39.5	38.9		38.8		37.7		38.2		39.5		39.3	
Hazardous Alcohol Use	60.5	61.1		61.2		62.3		61.9		60.5		60.8	
Substance Use^a^, %													
None	8.1		20.3		23.4		21.2		28.1		22.7		15.3
Drug Related Problems	91.9		79.7		76.3		78.8		71.9		77.3		84.7
Anxiety Symptoms, %													
None	9.6	14.1	15.3	15.3	16.6	17.2	17.4	18.0	20.1	19.7	20.2	20.7	20.0
Mild	25.5	31.2	29.8	31.3	31.1	30.2	28.3	31.5	32.8	31.7	31.9	31.2	30.2
Moderate	27.3	26.6	26.5	24.9	24.1	24.6	26.5	25.0	21.9	22.8	22.8	22.5	21.2
Severe	37.6	28.1	28.4	28.5	28.2	28.0	27.8	25.4	25.2	25.8	25.1	25.7	28.5
Depressive Symptoms, %													
None	5.4	7.6	7.1	9.1	10.1	10.5	11.3	11.6	13.0	12.0	13.1	13.8	12.6
Mild	16.4	20.8	23.5	23.3	23.3	23.7	23.2	25.8	25.9	26.7	28.1	26.6	23.4
Moderate	22.6	26.4	24.6	24.8	25.2	23.0	24.1	23.3	24.3	23.8	21.7	23.3	24.1
Moderately Severe	25.3	23.3	21.8	22.1	19.5	21.9	19.6	19.9	18.5	18.0	19.3	17.8	19.9
Severe	30.4	21.8	23.0	20.6	21.9	20.8	21.8	19.3	18.3	19.6	17.8	18.5	19.9

The following standardized cut-off values were used: AUDIT-C (Alcohol use) [58]: 3; DUDIT-C (Substance use) [59]: 1; GAD-7 (Anxiety symptoms) [60]: 4, 9, 14; and PHQ-9 (Depressive symptoms) [36]: 4, 9, 14, 19.

*AUDIT-C*, Alcohol Use Disorder Identification Test-Consumption; *B*, Baseline; *DUDIT-C*, Drug Use Disorder Identification Test-Consumption; *GAD-7*, Generalized Anxiety Disorder-7; *PHQ-9*, Patient Health Questionnaire-9.

^a^The follow-up used the short-form versions of the Drug Use Disorder Identification Test and the Alcohol Use Disorder Identification Test, which have a reduced number of cut-off values. For easier comparison over time, the short-form versions are also shown for the baseline timepoint in this table.

Eligible participants were aged 18 years or older and self-reported a current or past psychiatric diagnosis from a mental health care setting aligned with the DSM-5 [31] (see Supplementary Results: Sample Characteristics). Exclusion criteria included the inability to provide informed consent or the provision of incomplete baseline data.

The sample size reflected all eligible individuals enrolled during the recruitment period in the parent longitudinal cohort; therefore, no a priori power analysis was conducted for the present analyses [32]. The study followed the STROBE reporting guideline and was approved by the Swedish Ethical Review Authority (Dnr: 2020-02798, 2021-00521, and 2022-00648-02). All participants provided informed consent. The authors assert that all procedures contributing to this work comply with the ethical standards of the relevant national and institutional committees on human experimentation and with the Helsinki Declaration of 1975, as revised in 2008.

### Measures

Prosocial behavior was assessed only at baseline, whereas anxiety and depressive symptoms were assessed monthly and alcohol and substance use were assessed every second month throughout the one-year follow-up period.

#### Prosocial behaviors

Prosocial behavior was assessed using the Prosocial Tendencies Measure (PTM) [25], which captures six context-dependent subtypes: altruistic (helping at a personal cost), anonymous (helping without revealing one’s identity), compliant (helping upon request), dire (helping during emergency situations), emotional (helping when affectively aroused), and public (helping in front of others). Responses were rated on a 1–5 Likert scale, and subscale scores were computed as standardized mean item values. See Supplementary Methods for details and psychometric properties.

#### Psychiatric symptoms

The main psychiatric symptom measures during baseline and follow-up included alcohol use (Alcohol Use Disorder Identification Test, AUDIT [33]), substance use (Drug Use Disorder Identification Test, DUDIT [34]), anxiety (Generalized Anxiety Disorder 7-item scale, GAD-7 [35]), and depression (Patient Health Questionnaire 9-item scale, PHQ-9 [36]). During follow-up, abbreviated versions of alcohol and substance use were used (AUDIT-C and DUDIT-C). See Supplementary Methods for details and psychometric properties.

An additional exploratory set of analyses of psychiatric symptoms assessed attention deficit hyperactivity disorder [37], obsessive–compulsive disorder [38], posttraumatic stress disorder [39], and problem gambling [40]. These additional symptom measures were included as exploratory outcomes because they were assessed only at baseline and not longitudinally, precluding analyses of symptom trajectories over time. The results of these analyses are reported in Supplementary Results: Extended Models.

#### Psychiatric diagnoses

Psychiatric diagnoses were self-reported at baseline, using DSM-5 categories [31]. Participants indicated prior lifetime diagnoses received in mental health care settings (e.g., alcohol or substance use, anxiety, depressive, or trauma-related disorders). Details on the operationalization of each diagnostic group are provided in Supplementary Results: Sample Characteristics.

### Statistical analysis

Analyses were performed in R (version 4.2.2); statistical significance was set at P < 0.05 (two-tailed); analysis code is publicly available: https://osf.io/9fp7y.

Cross-sectional analyses examined associations between individual differences in six prosocial subtypes and each psychiatric symptom domain at baseline. These associations were examined using multiple linear regression models in which each psychiatric symptom domain was entered as continuous outcome variable while adding all different prosocial subtypes simultaneously as predictors. To account for shared variance across symptom dimensions, the remaining psychiatric symptom measures were included as covariates. All models additionally adjusted for demographic confounders (age, gender, and education level) associated with individual differences in prosocial behavior [41, 42].

Longitudinal analyses examined whether baseline prosocial subtypes predicted one-year longitudinal symptom trajectories for alcohol use, substance use, anxiety, and depressive symptoms, respectively. Because the primary aim was to identify clinically meaningful subgroups of individuals with distinct longitudinal symptom trajectories, rather than estimate a single average trajectory across the cohort, a person-centered trajectory modelling approach was considered most appropriate. Latent class linear mixed models (LCMMs) were selected because they allow identification of subgroups of participants with distinct longitudinal symptom trajectories and have been widely applied in psychiatric trajectory research [43–45]. Importantly, in comparison with other well-established methods (e.g., latent class growth analysis), LCMMs account for individual variability *within* classes through the inclusion of both fixed and random effects. The models identified classes of participants with distinct symptom trajectories over 12 months (e.g., low or high symptom load; see Supplementary Methods: Longitudinal Analyses). The resulting trajectory-class variable was then entered as the outcome variable in logistic regression models (low symptom class as reference) with baseline prosocial subtypes as predictors. Similar to our cross-sectional analyses, all models adjusted for demographic covariates, as well as shared variance with trajectory class membership in the remaining psychiatric symptom domains.

Both cross-sectional and longitudinal analyses were subjected to sensitivity analyses to account for recruitment occurring during the COVID-19 pandemic. Specifically, two contextual variables were added to the models: COVID-19 infection severity and subjective loneliness (given that the pandemic imposed general restrictions on social interaction) [2, 46]. Further details regarding multiple testing and model specifications are provided in the Supplementary Methods.

Missing data for the baseline regression analyses were handled using complete-case analysis (i.e., a complete baseline assessment was required). In contrast, longitudinal trajectory models were estimated using all available outcome data for each participant, even if some data were missing.

## Results

### Baseline associations between prosocial behavior and psychiatric symptom domains

**Fig. 1:**
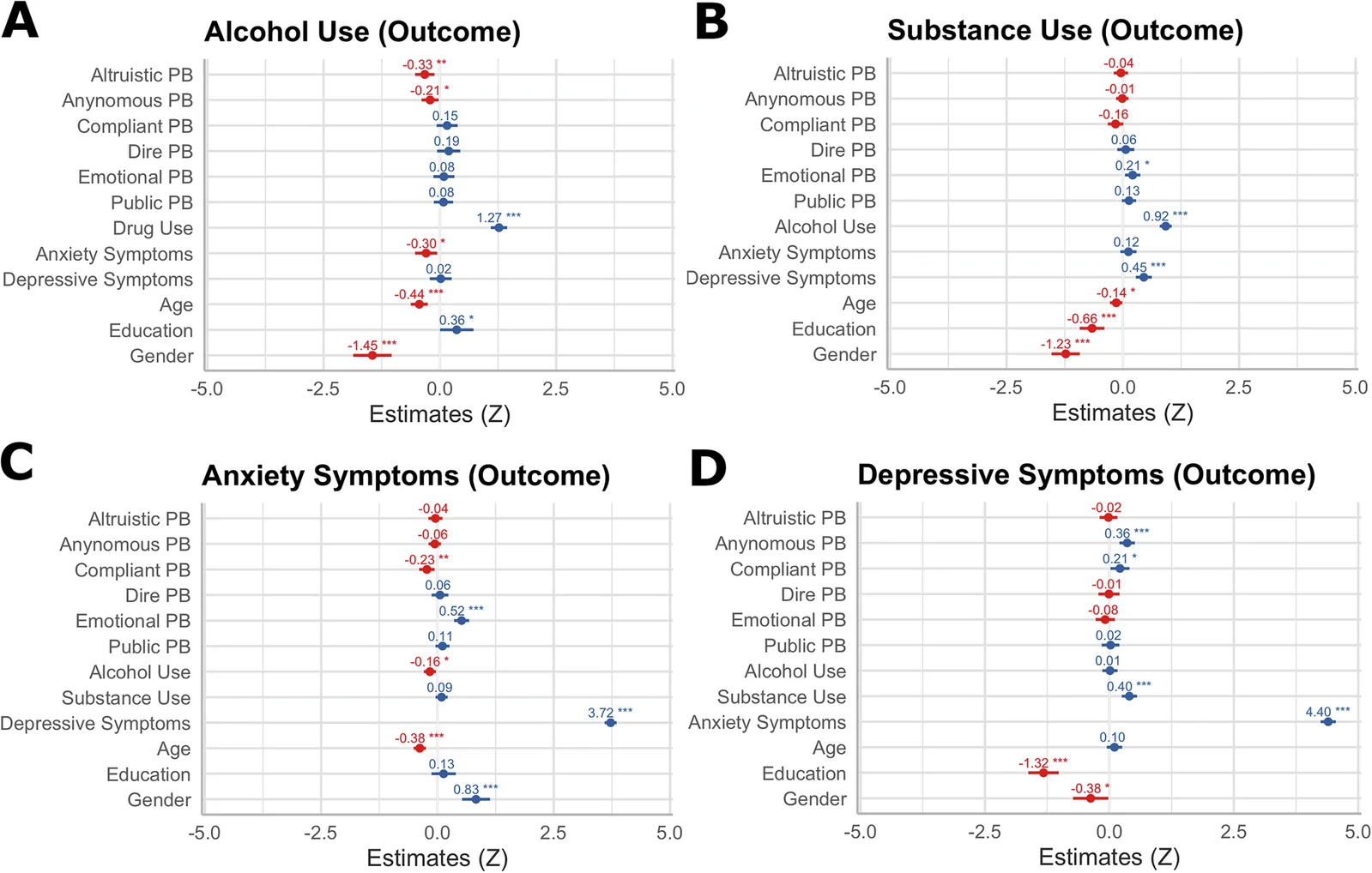
Baseline associations between psychiatric outcomes (A-D) and prosocial behaviors. Standardized regression coefficients (Z) from multiple linear regression models predicting (**A**) alcohol use, (**B**) substance use, (**C**) anxiety symptoms, and (**D**) depressive symptoms from six prosocial behavior subtypes (altruistic, anonymous, compliant, dire, emotional, and public prosocial behavior). Models additionally adjusted for shared variance across psychiatric symptom dimensions and demographic covariates (age, education, gender). Positive values indicate positive associations with the outcome; negative values indicate negative associations. For gender, positive values refer to females, whereas negative values refer to males. PB Prosocial Behavior.

We examined baseline associations between all prosocial behavior subtypes as predictors and each psychiatric symptom domain as outcome (Fig. 1). Lower altruistic prosociality was associated with higher alcohol use (β = –0.33, *p* = 0.002). Lower anonymous prosociality showed a similar association with higher alcohol use (β = –0.21, *p* = 0.023). In contrast, higher anonymous prosociality was associated with higher depressive symptoms (β = 0.37, *p* < 0.001).

Higher compliant prosociality was associated with higher depressive symptoms (β = 0.21, *p* = 0.030), but not when controlling for contextual factors during COVID-19 (infection severity and subjective loneliness, β = 0.16, *p* = 0.075).

Higher emotional prosociality was associated with higher substance use (β = 0.21, *p* = 0.013) and anxiety symptoms (β = 0.52, *p* < 0.001).

Dire and public prosociality respectively, showed no significant associations with any of the psychiatric symptom domains.

The effect sizes of the significant prosocial subtypes on psychiatric symptoms were small, but comparable to established clinical predictors [e.g., baseline associations reflecting standardized regression-based Cohen’s *d* equivalents ranging from 0.06 to 0.33], see Supplementary Results for details.

### Longitudinal associations between prosocial behavior and symptom trajectories

We next derived symptom trajectories over the 12-month period, followed by examining whether distinct prosocial behavior subtypes at baseline predicted longitudinal trajectories of psychiatric symptoms. Full trajectory and model results are presented in Supplementary Methods and Results: Longitudinal Analyses.

#### Alcohol use

**Fig. 2:**
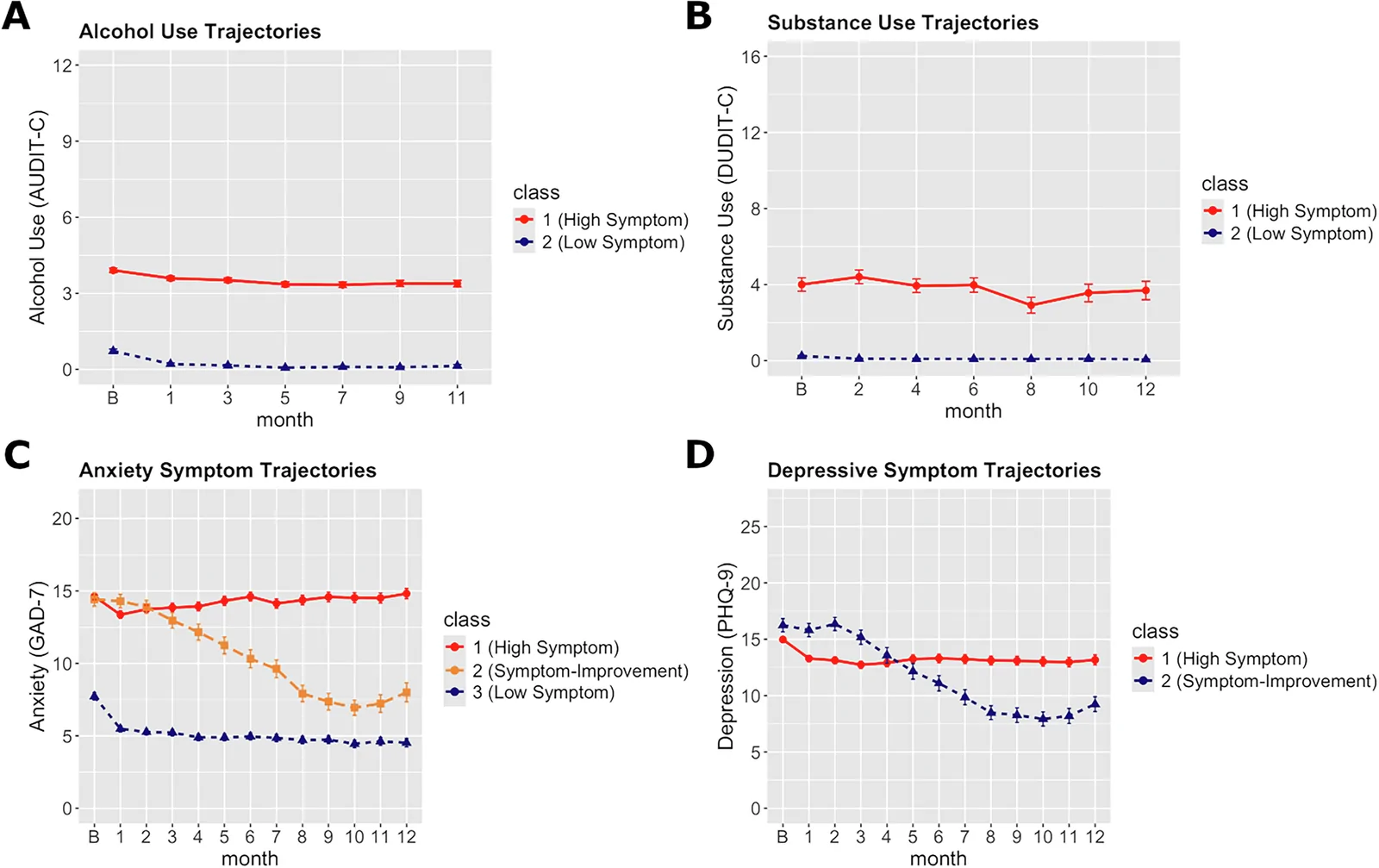
Psychiatric symptom trajectories across the 1-year follow-up period. Estimated mean symptom trajectories as derived from separate latent class linear mixed models for (**A**) alcohol use, (**B**), substance use, (**C**) anxiety symptoms, and (**D**) depressive symptoms. Lines denote predicted average symptom change for each class with error bars representing 95% confidence intervals. AUDIT-C (A) and DUDIT-C (B) values at baseline were transformed from the full scales (AUDIT and DUDIT, respectively) to match the interpretability of the longitudinal assessment. AUDIT Alcohol Use Disorder Identification Test; DUDIT Drug Use Disorder Identification Test; GAD-7 Generalized Anxiety Disorder-7; PHQ-9 Patient Health Questionnaire-9.

Two trajectory classes were identified for alcohol use: a stable high-symptom class (68.1%, *n* = 2103, Class 1) and a stable low-symptom class (31.9%, *n* = 989, Class 2), (Fig. 2A).

Higher levels of anonymous prosociality were associated with lower odds of belonging to the high symptom trajectory class (OR = 0.89, 95% CI = 0.82–0.98, *p* = 0.013).

#### Substance use

Two trajectory classes were identified for substance use: a stable high-symptom class (13.2%, *n* = 376, Class 1) and a stable low-symptom class (86.8%, *n* = 2464, Class 2) (Fig. 2B).

Higher levels of public prosociality were associated with higher odds of belonging to the high symptom trajectory relative to the low symptom trajectory (OR = 1.15, 95% CI = 1.01–1.31, *p* = 0.036).

#### Anxiety symptoms

Three trajectory classes were identified for anxiety symptoms: a stable high-symptom class (49.7%, *n* = 1622, Class 1), a symptom-improvement class (11.3%, *n* = 374, Class 2), and a stable low-symptom class (39.0%, *n* = 1282, Class 3), Fig. 2C.

In multinomial logistic regression models predicting anxiety trajectories, higher emotional prosociality was associated with increased odds of belonging to the stable high anxiety symptom class relative to the low anxiety symptom class (OR = 1.25, 95% CI = 1.12–1.40, p < 0.001). Higher emotional and public prosociality were also associated with increased odds of belonging to the symptom-improvement class relative to the low symptom class (OR = 1.22, 95% CI = 1.04–1.45, *p* = 0.017, and OR = 1.19, 95% CI = 1.01–1.39, *p* = 0.037, respectively). However, these associations became non-significant after adjusting for contextual factors related to the COVID-19 pandemic (infection severity and subjective loneliness; see Supplementary Results: Longitudinal Analyses).

#### Depressive symptoms

Two trajectory classes were identified for depressive symptoms: a stable high-symptom class (87.5%, *n* = 2876, Class 1) and a symptom-improvement class (12.5%, *n* = 410, Class 2) (Fig. 2D). There was no association between prosocial subtypes and subsequent depression symptom trajectories (Supplementary Results: Longitudinal Analyses).

### Sensitivity analyses

Sensitivity analyses adjusting for pandemic-related contextual factors (COVID-19 infection severity and subjective loneliness) yielded similar results for all baseline and longitudinal associations reported in the main manuscript, except where explicitly noted above. See Supplementary Results for details.

## Discussion

In the present study, we examined baseline and longitudinal associations between six prosocial behavior subtypes and a spectrum of psychiatric symptom domains in a large Swedish sample comprising individuals with diverse psychiatric disorders. Two key findings emerged. First, at baseline, prosocial behavior varied across all symptom domains examined, i.e., alcohol use, substance use, anxiety, and depressive symptoms. This highlights that prosociality is not a uniform construct in clinical populations. For example, lower levels of altruistic and anonymous prosocial behavior (helping at a personal cost, and helping without revealing one’s identity, respectively) were independently associated with higher alcohol use, while higher levels of emotional prosociality (helping when affectively aroused) were associated with symptoms of anxiety. Second, longitudinal analyses partially mirrored the baseline associations and identified three subtype-specific prospective associations with psychiatric symptom trajectories. Specifically: (1) lower anonymous prosocial behavior was associated with the persistent high alcohol use, (2) higher public prosociality was associated with the persistent high substance use, and (3) higher emotional prosociality was associated with persistent high anxiety symptoms. Together, the results of the study suggest that context-dependent forms of prosocial behavior may serve as informative behavioral markers of symptom persistence, across key psychiatric domains.

More broadly, the present findings support the notion that prosocial behavior may be relevant to mental health because it reflects how individuals engage with others. Previous research shows that prosocial behaviors contribute to the formation and maintenance of social relationships [7] and cooperation [6], both of which have been linked to psychiatric outcomes [2, 47]. From this perspective, context-dependent prosocial behaviors may represent behavioral manifestations of broader social processes that either promote resilience or contribute to the persistence of psychiatric symptoms. This interpretation is also consistent with emerging models of social homeostasis in psychiatry, including the social homeostasis model of addiction [47, 48], which propose that individuals regulate social behavior to maintain an optimal level of social connectedness, and that disruptions in this regulation may contribute to psychopathology, including harmful substance use. Importantly, our findings suggest that prosocial behaviors are not uniformly beneficial or maladaptive but depend on the specific social context in which helping behavior occurs.

Anonymous prosocial behavior has previously been demonstrated to be reduced following recreational cannabis use in healthy individuals [26]. Although we did not assess cannabis use specifically, our findings extend this work within alcohol use, showing that lower anonymous prosociality was associated with a higher alcohol use symptom trajectory in a psychiatric population. The lower propensity for anonymous helping among individuals with risky alcohol use may reflect greater self-referential motivational processes. This interpretation is consistent with prior research reporting a higher prevalence of antisocial traits [49] and selfish choices [11] in AUD.

Public prosocial behavior in healthy individuals has previously been predictive of later substance use [26] and associated with anxiety and depressive symptoms [27]. These findings are strengthened by our results in a psychiatric population by associating public prosociality with a higher substance use trajectory during the follow-up period. However, there was no association between public prosociality and baseline levels of substance use, which suggests that public prosociality is not a consequence or correlate of substance use but may represent a longitudinal vulnerability marker in seeking the approval and external validation of others.

Emotional prosocial behavior refers to helping others in emotionally arousing or distressing circumstances. In non-clinical samples, this prosocial subtype has been associated to both general anxiety symptoms [17, 27] and acute anxiety in threatening situations [50]. Extending these findings to a clinical population, the results of the current study found that higher emotional prosociality was associated with greater baseline anxiety and predicted higher anxiety trajectories over time. This pattern may reflect heightened emotional responsivity and empathic concern, which can exacerbate anxiety levels via interpersonal over-involvement [51]. Clinically, interventions that promote more regulated helping responses have the potential of reducing the risk of persistent anxiety symptoms.

Our findings also corroborate previous studies on altruism, reporting lower altruistic behavior among individuals with AUD and SUD compared to healthy individuals [11, 12]. While our study demonstrated baseline associations between altruism and alcohol as well as substance use, there were no longitudinal associations with later symptom development. This supports recent studies [13] suggesting a limited role of lower altruism as a risk factor for SUD. Interestingly, public prosociality showed a moderate correlation with altruistic prosociality (*r* = 0.55), and independently predicted subsequent substance use symptoms. This suggests that the associations observed between *reduced* altruism in SUD in previous studies may, in part, reflect shared variance with “socially visible helping behavior”, such as public prosociality.

Our results do not support that prosocial subtypes predict depressive symptom trajectories, although at baseline, higher levels of anonymous prosocial behavior were associated with greater depressive symptoms. This contrasts with a previous study that found higher prosociality to increase the vulnerability for later depressive symptoms, potentially, through excessive caregiving strains [29]. One possibility is that previous findings linking prosociality to depression are better explained by anxiety-related processes. Consistent with this interpretation, anxiety and depressive symptoms showed strong reciprocal associations in our models, and emotional prosociality demonstrated robust baseline and longitudinal associations with anxiety symptoms. Thus, the baseline association with depressive symptoms may partly reflect shared variance with anxiety rather than a depression-specific relationship. Alternatively, it may reflect state-dependent effects of depressed mood on self-reported prosocial tendencies (e.g., mood-congruent reporting) rather than stable trait-like effects of prosocial behavior on depressive symptoms over time.

Our study has some limitations. First, data collection occurred during the COVID-19 pandemic, a period that likely influenced social behavior and reduced opportunities for prosocial engagement [32, 52]. However, pandemic-related social restrictions in Sweden were relatively mild compared to most other countries [53]. Moreover, the observed associations between context-dependent prosocial behaviors and psychiatric symptom trajectories remained robust across multiple symptom domains, suggesting that these tendencies may reflect relatively stable behavioral dispositions rather than purely situational effects. In addition, sensitivity analyses adjusting for pandemic-related contextual factors, including subjective loneliness and COVID-19 infection severity, did not alter the main findings. The robustness of our results is consistent with previous conceptualizations of prosociality as a dispositional factor with implications for social functioning and mental health [3, 4].

Second, prosocial behavior was only assessed at baseline and was not repeatedly measured during the follow-up period. Consequently, although baseline prosocial behaviors predicted subsequent psychiatric symptom trajectories, we were unable to examine how prosociality changed over time or whether changes in prosocial behavior covaried with changes in psychiatric symptoms. While the prospective design strengthens temporal inference, it does not permit strong causal conclusions regarding the directionality of these associations. Future studies incorporating repeated assessments of both prosocial behavior and psychiatric symptoms may help clarify reciprocal influences and strengthen conclusions regarding potential causal pathways.

Third, the study used self-report measures of social behaviors which are sensitive to social desirability bias (i.e., a tendency to report what is socially expected) in psychiatric disorders [54]. However, given that we did find consistent effects, we believe that these would be stronger and more reliable using non-biased measures. For instance, the field of computational psychiatry has recently developed objective behavioral task measures targeting the mechanisms underlying social dysfunctions in psychiatric disorders [11, 55, 56], which might provide a promising avenue for future research on context-dependent prosocial behavior.

Finally, the trajectory classes were derived using a person-centered latent class modelling approach. Although this approach is well suited for identifying heterogeneous symptom trajectories, the resulting class solutions may be influenced by sample characteristics and modelling decisions and therefore require replication in independent cohorts.

In conclusion, individuals with heterogeneous psychiatric symptom profiles showed both shared and subtype-specific associations in prosocial behaviors defined by different social contexts. Notably, we identified three prosocial subtypes associated with later psychiatric symptom patterns: lower anonymous prosociality and elevated alcohol use, higher public prosociality and elevated substance use, and higher emotional prosociality and elevated anxiety symptoms. Importantly, prosociality was not uniformly reduced or increased; instead, different subtypes showed opposite associations with psychopathology. This pattern is consistent with classical accounts of moderation rooted in the Aristotelian doctrine of the mean (Aristotle, *Nichomachean Ethics*), suggesting that an intermediate level of social engagement may reflect a healthy balance between extremes such as social withdrawal and interpersonal overinvolvement [57]. Taken together, these findings contribute to transdiagnostic models of social dysfunction by supporting the view that prosocial behavior is context sensitive and may function as an index of persistent psychiatric symptom patterns.

Future studies should consider developing objective measures of prosocial subtypes in a clinical context using experimental and computational methodologies [11, 55, 56]. Another avenue for future research would be to test novel psychological treatment modules aimed at promoting or reducing identified deficits in subtypes of prosocial behavior and assess their effects on treatment outcomes.

## Supplementary information

Supplementary Information

## Data Availability

Analysis code is publicly available at: https://osf.io/9fp7y. The data supporting the findings of this study are not publicly available because they contain sensitive participant information and are subject to ethical restrictions. Requests for access to de-identified data will be considered on a case-by-case basis, subject to approval by the relevant ethical review authority and applicable data-sharing agreements.
