# Supplementary material for: Distinct prosocial behaviors associated with persistent symptom trajectories in psychiatric disorders
Source: Transl Psychiatry. 2026 Oct 5;16:522. doi: 10.1038/s41398-026-04515-3 (PMC13639033; doi:10.1038/s41398-026-04515-3)
Supplement: Supplementary file 1 [file 41398_2026_4515_MOESM1_ESM.docx]

**Distinct Prosocial Behaviors Associated with Persistent Symptom Trajectories in Psychiatric Disorders**

Supplementary Information

Table of Contents

[Supplementary Methods	3](#_Toc232687676)

[Dataset and Recruitment Procedure	3](#_Toc232687677)

[Multiple Testing	3](#_Toc232687678)

[Lifetime Self-Reported Psychiatric Diagnoses	4](#_Toc232687679)

[Longitudinal Analyses	5](#_Toc232687680)

[The Prosocial Tendencies Measure	6](#_Toc232687681)

[Psychiatric Symptom Measures	8](#_Toc232687682)

[Additional Exploratory Psychiatric Symptom Measures	12](#_Toc232687683)

[COVID-19-Related Measures	14](#_Toc232687684)

[Internal Consistency of the Prosocial Tendencies Measure	15](#_Toc232687685)

[Internal Consistency for the Psychiatric Symptom Measures	16](#_Toc232687686)

[Supplementary Results	17](#_Toc232687687)

[Sample Characteristics	17](#_Toc232687688)

[Demographics	18](#_Toc232687689)

[Baseline Analyses: Main Models	19](#_Toc232687690)

[Baseline Analyses: Extended Models	21](#_Toc232687691)

[Baseline Analyses: Effect Sizes	23](#_Toc232687692)

[Baseline Analyses: Exploratory Models (ADHD; OCD; PTSD; Gambling)	28](#_Toc232687693)

[Longitudinal Analyses: Main Models	32](#_Toc232687694)

[Longitudinal Analyses: Extended Models	37](#_Toc232687695)

[Longitudinal Analyses: Trajectory Characteristics	41](#_Toc232687696)

[Supplementary References	45](#_Toc232687697)

# Supplementary Methods

## Dataset and Recruitment Procedure

The present study used data from a larger longitudinal cohort study examining mental health trajectories during and following the COVID-19 pandemic in individuals with current or previous psychiatric disorders ^1^. Participants were informed that the study aimed to investigate psychiatric symptoms and social functioning over time.

Recruitment was conducted between July 2020 and June 2021 through outpatient psychiatric clinics across Sweden, social media platforms, online advertisements, patient organizations, and other digital channels. Interested individuals accessed study information online and provided electronic informed consent before completing the baseline assessment through the REDCap platform hosted by Karolinska Institutet.

Eligible participants were adults (≥18 years) residing in Sweden who reported a current or previous psychiatric disorder diagnosed within a healthcare setting. Participants completed a comprehensive baseline assessment covering psychiatric symptoms, social functioning, and related psychological constructs, followed by monthly follow-up assessments for one year.

Participants did not receive financial compensation for participation. Data collection was conducted entirely online to facilitate nationwide participation during the COVID-19 pandemic. Additional details regarding recruitment procedures, inclusion criteria, data collection, and cohort characteristics are provided in Rozental et al. (2022).

## Multiple Testing

Given the number of outcomes and predictors examined, we considered the issue of multiple testing. Formal adjustment for multiple comparisons was not deemed necessary, given that our approach was hypothesis-driven and included modelling each psychiatric domain within a multivariable model with all prosociality subtypes and remaining psychiatric domains as predictors.

To facilitate interpretation, we report exact p-values, confidence intervals, and effect sizes where appropriate. Results from exploratory analyses should be interpreted cautiously and considered hypothesis-generating.

## Lifetime Self-Reported Psychiatric Diagnoses

Lifetime psychiatric diagnoses from a mental healthcare setting were assessed at baseline by asking participants to confirm a specific disorder from a pre-defined list following the categorization in the fifth edition of the *Diagnostic and Statistical Manual of Mental Disorders* (DSM-5) ². Frequencies of all self-reported diagnoses are presented in Supplementary Table S4.

## Longitudinal Analyses

Here we investigated whether prosocial behavior at baseline could predict differences in subsequent psychiatric symptom load trajectories. Given that psychiatric symptom trajectories are heterogeneous and non-linear, we conducted **latent class linear mixed models** implemented using the R package lcmm ^2^. Models were estimated using maximum likelihood procedures and used to classify individuals into latent classes based on differences in the development of psychiatric symptom load over time (e.g., low vs high symptom load).

For each psychiatric symptom domain, we fitted latent class linear mixed models with an increasing number of classes. Multi-class models were estimated using a grid-search procedure with 200 random starts and 100 iterations to reduce the risk of convergence on local maxima. Model selection was based on a combination of statistical fit and clinical interpretability. The Bayesian Information Criterion (BIC) was used as the primary fit index because it balances model fit and model complexity and has been shown to perform well for class enumeration in latent class and mixture models ^3^. In addition, we required each retained class to include at least 10% of participants to avoid unstable or clinically difficult-to-interpret small classes and to avoid unstable or difficult-to-interpret small classes. Thus, the retained model was the model with the lowest BIC among solutions in which all classes included at least 10% of participants. Model-selection indices for each outcome are reported in Supplementary Table S1.

| **Supplementary Table S1.** Model selection indices for latent class linear mixed models of psychiatric symptom trajectories. | | | | | | |
| --- | --- | --- | --- | --- | --- | --- |
| **Outcome** | **1-class BIC** | **2-class BIC** | **2-class proportions** | **3-class BIC** | **3-class proportions** | **Retained** |
| Alcohol use | 41031.37 | 40118.35 | 31.9%, 68.1% | 39596.91 | 32.2%, 3.7%, 64.1% | 2-class |
| Substance use | 36455.44 | 30810.19 | 86.8%, 13.2% | 29028.21 | 84.8%, 9.5%, 5.6% | 2-class |
| Anxiety | 131939.80 | 131295.50 | 10.4%, 89.6% | 130988.50 | 49.7%, 11.3%, 39.0% | 3-class |
| Depression | 138855.40 | 138179.60 | 87.4%, 12.6% | 137913.80 | 10.2%, 85.1%, 4.7% | 2-class |
| BIC = Bayesian Information Criterion. The retained model was the model with the lowest BIC among solutions in which all latent classes included at least 10% of participants. | | | | | | |

Following the classification of individuals into different classes for each psychiatric domain, we applied a logistic regression model using the classification variable as a dependent variable and adding the six prosocial subtypes and demographic variables (age, gender, education) as independent variables. All models adjusted for shared variance with trajectory class membership in the remaining psychiatric symptom domains.

## The Prosocial Tendencies Measure

Subscale definitions:

The Prosocial Tendencies Model is based on behavioral observations of different helping opportunities and proposed six subtypes of context-dependent prosocial behaviors:

- altruistic (helping without the expectation for self-rewards),

- anonymous (helping without revealing one’s identity),

- compliant (helping upon request),

- dire (helping during emergency situations),

- emotional (helping in affectively arousing situations), and

- public (helping in front of others).

The Prosocial Tendencies Measure, instructions and items per subscale:

Altruistic prosocial behavior (reversely coded)

- I think that one of the best things about helping others is that it makes me look good.

- I believe that donating goods or money works best when it is tax-deductible.

- I think there should be more recognition for the time and energy people spend on charity work.

- One of the best things about doing charity work is that it looks good on my resume.

- I feel that if I help someone, they should help me in the future.

Anonymous prosocial behavior

- I prefer to donate money anonymously.

- I tend to help needy others most when they do not know who helped them.

- Most of the time, I help others when they do not know who helped them.

- I think that helping others without them knowing is the best type of situation.

- I often make anonymous donations because they make me feel good.

Compliant prosocial behavior

- When people ask me to help them, I don't hesitate.

- I never hesitate to help others when they ask for it.

Dire prosocial behavior

- I tend to help people who are in a real crisis or need.

- I tend to help people who hurt themselves badly.

- It is easy for me to help others when they are in a dire situation.

Emotional prosocial behavior

- It is most fulfilling to me when I can comfort someone who is very distressed.

- I tend to help others particularly when they are emotionally distressed.

- I respond to helping others best when the situation is highly emotional.

- Emotional situations make me want to help needy others.

Public prosocial behavior

- I can help others best when people are watching me.

- When other people are around, it is easier for me to help needy others.

- I get the most out of helping others when it is done in front of others.

- Helping others when I am in the spotlight is when I work best.

Responses were rated on a 1-5 Likert scale:

*Below are a number of statements which may or may not describe you.*

*Please indicate HOW MUCH EACH STATEMENT DESCRIBES YOU by using the scale below:*

*1 Does not describe me at all*

*2 Describes me a little*

*3 Somewhat describes me*

*4 Describes me well*

*5 Describes me greatly*

## Psychiatric Symptom Measures

**Alcohol Use (AUDIT).** Alcohol use was assessed using the 10-item Alcohol Use Disorders Identification Test (AUDIT) ^4^. Responses are summed to produce a total score ranging from 0–40, with higher scores indicating greater alcohol-related problems.

Response options:

Items 1–8:
0 = Never
1 = Less than monthly
2 = Monthly
3 = Weekly
4 = Daily or almost daily

Items 9–10:
0 = No
2 = Yes, but not in the last year
4 = Yes, during the last year

Scale items:

1. How often do you have a drink containing alcohol?
2. How many drinks containing alcohol do you have on a typical day when you are drinking?
3. How often do you have six or more drinks on one occasion?
4. How often during the last year have you found that you were not able to stop drinking once you had started?
5. How often during the last year have you failed to do what was normally expected of you because of drinking?
6. How often during the last year have you needed a first drink in the morning to get yourself going after a heavy drinking session?
7. How often during the last year have you had a feeling of guilt or remorse after drinking?
8. How often during the last year have you been unable to remember what happened the night before because of drinking?
9. Have you or someone else been injured because of your drinking?
10. Has a relative, friend, doctor, or other health worker been concerned about your drinking or suggested that you cut down?

**Substance Use (DUDIT).** Substance use was assessed using the 11-item Drug Use Disorders Identification Test (DUDIT) ^5^**.** Responses are summed to produce a total score ranging from 0–44, with higher scores indicating greater drug-related problems.

Response options:

Items 1–9:
0 = Never
1 = Less than monthly
2 = Monthly
3 = Weekly
4 = Daily or almost daily

Items 10–11:
0 = No
2 = Yes, but not in the past year
4 = Yes, during the past year

Scale items:

1. How often do you use drugs other than alcohol?
2. Do you use more than one type of drug on the same occasion?
3. How often are you under the influence of drugs?
4. During the past year, how often have you felt that your longing for drugs was so strong that you could not resist it?
5. During the past year, has it happened that you have not been able to stop using drugs once you started?
6. During the past year, how often have you taken drugs and then neglected to do something you should have done?
7. During the past year, how often have you needed to take a drug the morning after heavy drug use the day before?
8. During the past year, how often have you had feelings of guilt or a bad conscience because you used drugs?
9. How often have you or someone else been hurt (mentally or physically) because you used drugs?
10. Has a relative, friend, nurse, doctor, or anyone else been worried about your drug use or told you to stop using drugs?
11. Have you ever experienced problems because of your drug use?

**Anxiety Symptoms (GAD-7).** Anxiety symptoms were assessed using the Generalized Anxiety Disorder-7 scale (GAD-7) ^6^. Responses are summed to produce a total score ranging from 0–21.

Response options:

0 = Not at all
1 = Several days
2 = More than half the days
3 = Nearly every day

Scale items:

Over the last two weeks, how often have you been bothered by the following problems?

1. Feeling nervous, anxious, or on edge
2. Not being able to stop or control worrying
3. Worrying too much about different things
4. Trouble relaxing
5. Being so restless that it is hard to sit still
6. Becoming easily annoyed or irritable
7. Feeling afraid as if something awful might happen

**Depressive Symptoms (PHQ-9).** Depressive symptoms were assessed using the Patient Health Questionnaire-9 (PHQ-9) ^7^. Responses are summed to produce a total score ranging from 0–27.

Response options:

0 = Not at all
1 = Several days
2 = More than half the days
3 = Nearly every day

Scale items:

Over the last two weeks, how often have you been bothered by any of the following problems?

1. Little interest or pleasure in doing things
2. Feeling down, depressed, or hopeless
3. Trouble falling or staying asleep, or sleeping too much
4. Feeling tired or having little energy
5. Poor appetite or overeating
6. Feeling bad about yourself — or that you are a failure or have let yourself or your family down
7. Trouble concentrating on things, such as reading the newspaper or watching television
8. Moving or speaking so slowly that other people could have noticed? Or the opposite — being so fidgety or restless that you have been moving around a lot more than usual
9. Thoughts that you would be better off dead or of hurting yourself in some way

## Additional Exploratory Psychiatric Symptom Measures

An additional set of psychiatric symptom measures was assessed at baseline for exploratory analyses only, as these measures were not repeatedly assessed during follow-up and therefore could not be included in the longitudinal trajectory analyses.

**Attention Deficit Hyperactivity Disorder (BAARS-IV)**

ADHD symptoms were assessed using the 18-item Barkley Adult ADHD Rating Scale-IV (BAARS-IV) ^8^. The scale assesses symptoms of inattention and hyperactivity/impulsivity based on DSM criteria. Responses are rated on a 0–3 Likert scale:

0 = Never or rarely
1 = Sometimes
2 = Often
3 = Very often

Example items include:

- Difficulty sustaining attention in tasks or activities.
- Avoids or dislikes tasks that require sustained mental effort.
- Fidgets with hands or feet or squirms in seat.
- Interrupts or intrudes on others.

Responses are summed to produce a total symptom score, with higher scores indicating greater ADHD symptom severity.

**Obsessive–Compulsive Disorder (OCI-R)**

Obsessive–compulsive symptoms were assessed using the 18-item Obsessive–Compulsive Inventory–Revised (OCI-R) ^9^. The scale assesses six symptom dimensions: washing, checking, ordering, obsessing, hoarding, and neutralizing. Responses are rated on a 0–4 Likert scale:

0 = Not at all
1 = A little
2 = Moderately
3 = A lot
4 = Extremely

Example items include:

- I check things more often than necessary.
- I wash my hands more often and longer than necessary.
- I am upset by unpleasant thoughts that come into my mind against my will.
- I need things to be arranged in a particular order.

Responses are summed to produce a total symptom score, with higher scores indicating greater obsessive–compulsive symptom severity.

**Posttraumatic Stress Disorder (PCL-5)**

PTSD symptoms were assessed using the PTSD Checklist for DSM-5 (PCL-5), ^10^. The scale assesses symptoms corresponding to DSM-5 PTSD criteria over the past month. Responses are rated on a 0–4 Likert scale:

0 = Not at all
1 = A little bit
2 = Moderately
3 = Quite a bit
4 = Extremely

Example items include:

- Repeated, disturbing, and unwanted memories of the stressful experience.
- Avoiding memories, thoughts, or feelings related to the stressful experience.
- Feeling distant or cut off from other people.
- Being super-alert, watchful, or on guard.

Responses are summed to produce a total symptom score, with higher scores indicating greater PTSD symptom severity.

**Problem Gambling (PGSI)**

Problem gambling symptoms were assessed using the 9-item Problem Gambling Severity Index ^11^. The scale assesses gambling-related problems and adverse consequences during the previous 12 months. Responses are rated on a 0–3 Likert scale:

0 = Never
1 = Sometimes
2 = Most of the time
3 = Almost always

Example items include:

- Have you bet more than you could really afford to lose?
- Have you felt guilty about the way you gamble or what happens when you gamble?
- Has your gambling caused any health problems, including stress or anxiety?
- Has your gambling caused financial problems for you or your household?

Responses are summed to produce a total symptom score, with higher scores indicating greater gambling-related problems.

## COVID-19-Related Measures

**COVID-19 Infection.**

Given the timing of our data collection, we controlled for the severity of a potential COVID-19 infection in all our analyses. We used a self-made questionnaire (ranging from 0 to 3 points) to assess the potential symptom severity of a COVID-19 infection.

*Have you had confirmed COVID-19, and if so, indicate the severity?*

- *No*
- *Yes, I have had symptoms and stayed at home.*
- *Yes, I have been on sick leave due to COVID-19.*
- *Yes, I have needed hospital care.*

**Subjective Loneliness.**

Next, given that the pandemic imposed general restrictions on social interaction, we controlled for subjective loneliness using the 3-item UCLA Loneliness Scale, ranging from 1 (Rarely) to 2 (Always) ^12^:

*The following statements describe how people sometimes feel. For each statement, please indicate how often you feel the way described*

- *How often do you feel that you are “in tune” with the people around you?*
- *How often do you feel that no one really knows you well?*
- *How often do you feel you can find companionship when you want it?*
- *How often do you feel that people are around you but not with you?*

## Internal Consistency of the Prosocial Tendencies Measure

The subscales of the prosocial tendencies measure (PTM) have demonstrated adequate internal consistency, test-retest reliability, and convergent validity in previous studies ^13^, and demonstrate acceptable to good reliability in our study, in accordance with below:

| **Supplementary Table S2.** Internal consistence for PTM | | |
| --- | --- | --- |
| **Subscale** | **Number of Items** | **Cronbach's Alpha** |
| Altruistic Prosocial Behavior | 5 | 0.6307678 |
| Compliant Prosocial Behavior | 2 | 0.838859 |
| Dire Prosocial Behavior | 3 | 0.7922564 |
| Emotional Prosocial Behavior | 4 | 0.6910425 |
| Anonymous Prosocial Behavior | 5 | 0.7788728 |
| Public Prosocial Behavior | 4 | 0.7173894 |

## Internal Consistency for the Psychiatric Symptom Measures

The scales on psychiatric symptom load demonstrate good to excellent reliability in accordance with below:

| **Supplementary Table S3.** Internal consistence for the psychiatric symptom measures | | |
| --- | --- | --- |
| **Measure** | **Number of Items** | **Cronbach's Alpha** |
| AUDIT (Alcohol use) | 10 | 0.9054329 |
| DUDIT (Drug use) | 11 | 0.9288094 |
| GAD-7 (Anxiety symptoms) | 7 | 0.9040281 |
| PHQ-9 (Depressive symptoms) | 9 | 0.8822395 |
|  |  |  |
| BAARS-IV (ADHD symptoms) | 18 | 0.907144 |
| OCIR (OCD symptoms) | 18 | 0.8795631 |
| PCL-5 (PTSD) | 21 | 0.9514198 |
| PGSI (Gambling) | 9 | 0.9517733 |
| **Note:** Cronbach’s α values reflect internal consistency in the current study sample. For the PCL-5, internal consistency was estimated among participants endorsing trauma exposure.  **Abbreviations:** AUDIT = Alcohol Use Disorders Identification Test; DUDIT = Drug Use Disorders Identification Test; GAD-7 = Generalized Anxiety Disorder–7; PHQ-9 = Patient Health Questionnaire–9; BAARS-IV = Barkley Adult ADHD Rating Scale–IV; OCI-R = Obsessive–Compulsive Inventory–Revised; PCL-5 = PTSD Checklist for DSM-5; PGSI = Problem Gambling Severity Index. | | |

# Supplementary Results

## Sample Characteristics

Frequencies of all self-reported lifetime psychiatric diagnoses from a mental healthcare setting are presented in Supplementary Table S4.

| **Supplementary Table S4.** Lifetime self-reported psychiatric diagnoses from a mental healthcare setting at baseline (N = 4,484). | | |
| --- | --- | --- |
| **Diagnosis** | **n** | **Percent** |
| Major depressive disorder | 3491 | 77.9 |
| Generalized anxiety disorder | 2052 | 45.8 |
| Panic disorder | 1873 | 41.8 |
| Attention-deficit/hyperactivity disorder or autism spectrum disorder | 1068 | 23.8 |
| Post-traumatic stress disorder | 926 | 20.7 |
| Social anxiety disorder | 861 | 19.2 |
| Eating disorder | 733 | 16.3 |
| Bipolar disorder | 517 | 11.5 |
| Alcohol or substance use disorder | 414 | 9.2 |
| Obsessive-compulsive disorder | 395 | 8.8 |
| Hypomanic or manic depression | 315 | 7.0 |
| Illness anxiety disorder | 295 | 6.6 |
| Specific phobia | 213 | 4.8 |
| Anorexia nervosa | 211 | 4.7 |
| Agoraphobia | 199 | 4.4 |
| Psychotic disorder | 162 | 3.6 |
| Postpartum depression | 152 | 3.4 |
| Premenstrual dysphoric disorder | 129 | 2.9 |
| Do not know | 77 | 1.7 |
| Schizophrenia | 30 | 0.7 |

## Demographics

Bivariate correlations indicated small-to-moderate relationships among the six prosocial subtypes (Pearson *r* = .05 to .63; see Supplementary Figure S1).

**Supplementary Figure S1.** Correlations between psychiatric symptoms and subtypes of prosocial behavior at baseline. Values in each cell display the Pearson r coefficients.

**Abbreviations:** PB = Prosocial Behavior

## Baseline Analyses: Main Models

We created four multiple linear regression models, each including all six prosocial subtypes, as well as age, gender and education as independent variables, and for each model, one of the psychiatric scales as a dependent variable, while controlling for the remaining psychiatric symptom scales as independent variables. To simplify the interpretation of the results, we excluded data on gender where the participants not identified as male or female gender (n=231).

*Generic linear regression model specification:*

*psychScale1 ~ ptmAnynomousZ + ptmCompliantZ+ ptmDireZ+ ptmAltruismZ+ ptmEmotionZ+ ptmPublicZ + psychScale2Z + psychScale3Z + psychScale4Z + ageZ + education + gender*

| **Supplementary Table S5.** Baseline Alcohol use-demographics model | | | | |
| --- | --- | --- | --- | --- |
|  | Estimate | Std. Error | t value | Pr(>\|t\|) |
| (Intercept) | 9.85823 | 0.46174 | 21.350 | < 2e-16*** |
| ptmAltruismZ | -0.32552 | 0.10614 | -3.067 | 0.00218** |
| ptmAnynomousZ | -0.21201 | 0.09312 | -2.277 | 0.02285* |
| ptmCompliantZ | 0.15496 | 0.11497 | 1.348 | 0.17781 |
| ptmDireZ | 0.18883 | 0.12686 | 1.489 | 0.13669 |
| ptmEmotionZ | 0.08492 | 0.11484 | 0.739 | 0.45966 |
| ptmPublicZ | 0.07713 | 0.10585 | 0.729 | 0.46625 |
| DUDITZ | 1.26924 | 0.09001 | 14.102 | < 2e-16*** |
| GAD7Z | -0.29686 | 0.12064 | -2.461 | 0.01391* |
| PHQ9Z | 0.01738 | 0.12016 | 0.145 | 0.88498 |
| ageZ | -0.44313 | 0.09217 | -4.808 | 1.58e-06*** |
| education | 0.36159 | 0.18364 | 1.969 | 0.04901* |
| gender | -1.45088 | 0.21013 | -6.905 | 5.78e-12*** |
| Residual standard error: 5.697 on 4240 degrees of freedom  (231 observations deleted due to missingness)  Multiple R-squared: 0.0809, Adjusted R-squared: 0.0783  F-statistic: 31.1 on 12 and 4240 DF, p-value: < 2.2e-16 | | | | |

| **Supplementary Table S6.** Baseline Substance use-demographics model | | | | |  |
| --- | --- | --- | --- | --- | --- |
|  | Estimate | Std. Error | t value | Pr(>\|t\|) | |
| (Intercept) | 4.64237 | 0.33642 | 13.799 | < 2e-16*** | |
| ptmAltruismZ | -0.04285 | 0.07805 | -0.549 | 0.5831 | |
| ptmAnynomousZ | -0.01369 | 0.06844 | -0.200 | 0.8414 | |
| ptmCompliantZ | -0.15787 | 0.08444 | -1.870 | 0.0616. | |
| ptmDireZ | 0.06233 | 0.09320 | 0.669 | 0.5037 | |
| ptmEmotionZ | 0.20987 | 0.08430 | 2.489 | 0.0128* | |
| ptmPublicZ | 0.13153 | 0.07773 | 1.692 | 0.0907. | |
| AUDITZ | 0.92108 | 0.06532 | 14.102 | < 2e-16*** | |
| GAD7Z | 0.11861 | 0.08867 | 1.338 | 0.1811 | |
| PHQ9Z | 0.45025 | 0.08800 | 5.117 | 3.25e-07*** | |
| ageZ | -0.14182 | 0.06785 | -2.090 | 0.0367* | |
| education | -0.66254 | 0.13457 | -4.923 | 8.84e-07*** | |
| gender | -1.22808 | 0.15407 | -7.971 | 2.01e-15*** | |
| Residual standard error: 4.185 on 4240 degrees of freedom  (231 observations deleted due to missingness)  Multiple R-squared: 0.1015, Adjusted R-squared: 0.099  F-statistic: 39.93 on 12 and 4240 DF, p-value: < 2.2e-16 | | | | | |

| **Supplementary Table S7.** Baseline Anxiety-demographics model | | | | |
| --- | --- | --- | --- | --- |
|  | Estimate | Std. Error | t value | Pr(>\|t\|) |
| (Intercept) | 10.31234 | 0.33435 | 30.843 | < 2e-16*** |
| ptmAltruismZ | -0.04291 | 0.07698 | -0.557 | 0.57734 |
| ptmAnynomousZ | -0.05609 | 0.06751 | -0.831 | 0.40611 |
| ptmCompliantZ | -0.22659 | 0.08325 | -2.722 | 0.00652** |
| ptmDireZ | 0.05509 | 0.09194 | 0.599 | 0.54904 |
| ptmEmotionZ | 0.51936 | 0.08283 | 6.270 | 3.97e-10*** |
| ptmPublicZ | 0.10934 | 0.07668 | 1.426 | 0.15397 |
| AUDITZ | -0.16209 | 0.06587 | -2.461 | 0.01391* |
| DUDITZ | 0.08924 | 0.06671 | 1.338 | 0.18106 |
| PHQ9Z | 3.72231 | 0.06567 | 56.680 | < 2e-16*** |
| ageZ | -0.37956 | 0.06671 | -5.690 | 1.36e-08*** |
| education | 0.13362 | 0.13311 | 1.004 | 0.31551 |
| gender | 0.82875 | 0.15258 | 5.432 | 5.90e-08*** |
| Residual standard error: 4.128 on 4240 degrees of freedom  (231 observations deleted due to missingness)  Multiple R-squared: 0.4763, Adjusted R-squared: 0.4748  F-statistic: 321.4 on 12 and 4240 DF, p-value: < 2.2e-16 | | | | |

| **Supplementary Table S8.** Baseline Depression-demographics model | | | | |  |
| --- | --- | --- | --- | --- | --- |
|  | Estimate | Std. Error | t value | Pr(>\|t\|) | |
| (Intercept) | 18.00407 | 0.39191 | 45.940 | < 2e-16*** | |
| ptmAltruismZ | -0.01864 | 0.09048 | -0.206 | 0.8368 | |
| ptmAnynomousZ | 0.35634 | 0.07915 | 4.502 | 6.92e-06*** | |
| ptmCompliantZ | 0.21213 | 0.09787 | 2.167 | 0.0303* | |
| ptmDireZ | -0.00884 | 0.10805 | -0.082 | 0.9348 | |
| ptmEmotionZ | -0.08404 | 0.09779 | -0.859 | 0.3902 | |
| ptmPublicZ | 0.02267 | 0.09014 | 0.251 | 0.8015 | |
| AUDITZ | 0.01121 | 0.07747 | 0.145 | 0.8850 | |
| DUDITZ | 0.40001 | 0.07818 | 5.117 | 3.25e-07*** | |
| GAD7Z | 4.39515 | 0.07754 | 56.680 | < 2e-16*** | |
| ageZ | 0.10176 | 0.07868 | 1.293 | 0.1960 | |
| education | -1.32485 | 0.15512 | -8.541 | < 2e-16*** | |
| gender | -0.37698 | 0.17985 | -2.096 | 0.0361* | |
| Residual standard error: 4.851 on 4240 degrees of freedom  (231 observations deleted due to missingness)  Multiple R-squared: 0.4744, Adjusted R-squared: 0.4729  F-statistic: 319 on 12 and 4240 DF, p-value: < 2.2e-16 | | | | | |

## Baseline Analyses: Extended Models

**Controlling for COVID-19-related variables: COVID-19 infection and subjective loneliness.**

| **Supplementary Table S9.** Baseline Alcohol use-COVID model | | | | |
| --- | --- | --- | --- | --- |
|  | Estimate | Std. Error | t value | Pr(>\|t\|) |
| (Intercept) | 9.92953 | 0.46292 | 21.450 | < 2e-16*** |
| ptmAltruismZ | -0.32329 | 0.10613 | -3.046 | 0.00233** |
| ptmAnynomousZ | -0.20819 | 0.09319 | -2.234 | 0.02553* |
| ptmCompliantZ | 0.15776 | 0.11495 | 1.372 | 0.17001 |
| ptmDireZ | 0.17573 | 0.12738 | 1.380 | 0.16778 |
| ptmEmotionZ | 0.09094 | 0.11487 | 0.792 | 0.42859 |
| ptmPublicZ | 0.08114 | 0.10585 | 0.767 | 0.44338 |
| DUDITZ | 1.26841 | 0.09000 | 14.093 | < 2e-16*** |
| GAD7Z | -0.28194 | 0.12121 | -2.326 | 0.02006* |
| PHQ9Z | 0.09572 | 0.12823 | 0.746 | 0.45542 |
| ageZ | -0.43015 | 0.09239 | -4.656 | 3.32e-06*** |
| education | 0.36112 | 0.18378 | 1.965 | 0.04949* |
| gender | -1.49126 | 0.21116 | -7.062 | 1.91e-12*** |
| lonelinessZ | -0.16274 | 0.10210 | -1.594 | 0.11105 |
| covidInfectZ | -0.11772 | 0.08704 | -1.352 | 0.17630 |
| Residual standard error: 5.695 on 4238 degrees of freedom  (231 observations deleted due to missingness)  Multiple R-squared: 0.08182, Adjusted R-squared: 0.07879  F-statistic: 26.98 on 14 and 4238 DF, p-value: < 2.2e-16 | | | | |

| **Supplementary Table S10.** Baseline Substance use-COVID model | | | | |
| --- | --- | --- | --- | --- |
|  | Estimate | Std. Error | t value | Pr(>\|t\|) |
| (Intercept) | 4.63476 | 0.33747 | 13.734 | < 2e-16*** |
| ptmAltruismZ | -0.04461 | 0.07806 | -0.571 | 0.5677 |
| ptmAnynomousZ | -0.01023 | 0.06852 | -0.149 | 0.8813 |
| ptmCompliantZ | -0.15790 | 0.08445 | -1.870 | 0.0616. |
| ptmDireZ | 0.05345 | 0.09361 | 0.571 | 0.5680 |
| ptmEmotionZ | 0.21149 | 0.08435 | 2.507 | 0.0122* |
| ptmPublicZ | 0.12961 | 0.07776 | 1.667 | 0.0956. |
| AUDITZ | 0.92103 | 0.06535 | 14.093 | < 2e-16*** |
| GAD7Z | 0.12688 | 0.08910 | 1.424 | 0.1545 |
| PHQ9Z | 0.46840 | 0.09396 | 4.985 | 6.43e-07*** |
| ageZ | -0.14026 | 0.06803 | -2.062 | 0.0393* |
| education | -0.67025 | 0.13471 | -4.975 | 6.77e-07*** |
| gender | -1.21728 | 0.15495 | -7.856 | 4.99e-15*** |
| lonelinessZ | -0.05263 | 0.07504 | -0.701 | 0.4832 |
| covidInfectZ | 0.06860 | 0.06396 | 1.072 | 0.2836 |
| Residual standard error: 4.185 on 4238 degrees of freedom  (231 observations deleted due to missingness)  Multiple R-squared: 0.1019, Adjusted R-squared: 0.09893  F-statistic: 34.35 on 14 and 4238 DF, p-value: < 2.2e-16 | | | | |

| **Supplementary Table S11.** Baseline Anxiety-COVID model | | | | |
| --- | --- | --- | --- | --- |
|  | Estimate | Std. Error | t value | Pr(>\|t\|) |
| (Intercept) | 10.23954 | 0.33371 | 30.684 | < 2e-16*** |
| ptmAltruismZ | -0.03847 | 0.07664 | -0.502 | 0.61571 |
| ptmAnynomousZ | -0.07462 | 0.06726 | -1.109 | 0.26730 |
| ptmCompliantZ | -0.22863 | 0.08287 | -2.759 | 0.00583** |
| ptmDireZ | 0.10910 | 0.09190 | 1.187 | 0.23523 |
| ptmEmotionZ | 0.49823 | 0.08252 | 6.038 | 1.70e-09*** |
| ptmPublicZ | 0.10924 | 0.07635 | 1.431 | 0.15257 |
| AUDITZ | -0.15261 | 0.06561 | -2.326 | 0.02006* |
| DUDITZ | 0.09458 | 0.06642 | 1.424 | 0.15451 |
| PHQ9Z | 3.49342 | 0.07536 | 46.355 | < 2e-16*** |
| ageZ | -0.40118 | 0.06654 | -6.030 | 1.78e-09*** |
| education | 0.16365 | 0.13262 | 1.234 | 0.21728 |
| gender | 0.84521 | 0.15268 | 5.536 | 3.28e-08*** |
| lonelinessZ | 0.45742 | 0.07335 | 6.236 | 4.92e-10*** |
| covidInfectZ | -0.08553 | 0.06280 | -1.362 | 0.17324 |
| Residual standard error: 4.108 on 4238 degrees of freedom  (231 observations deleted due to missingness)  Multiple R-squared: 0.4814, Adjusted R-squared: 0.4797  F-statistic: 281 on 14 and 4238 DF, p-value: < 2.2e-16 | | | | |

| **Supplementary Table S12.** Baseline Depression-COVID model | | | | |
| --- | --- | --- | --- | --- |
|  | Estimate | Std. Error | t value | Pr(>\|t\|) |
| (Intercept) | 17.149436 | 0.369269 | 46.442 | < 2e-16*** |
| ptmAltruismZ | -0.013620 | 0.084793 | -0.161 | 0.872395 |
| ptmAnynomousZ | 0.248729 | 0.074322 | 3.347 | 0.000825*** |
| ptmCompliantZ | 0.163225 | 0.091729 | 1.779 | 0.075242. |
| ptmDireZ | 0.185629 | 0.101644 | 1.826 | 0.067882. |
| ptmEmotionZ | -0.140105 | 0.091659 | -1.529 | 0.126451 |
| ptmPublicZ | 0.005846 | 0.084488 | 0.069 | 0.944838 |
| AUDITZ | 0.054212 | 0.072623 | 0.746 | 0.455418 |
| DUDITZ | 0.365322 | 0.073280 | 4.985 | 6.43e-07*** |
| GAD7Z | 3.655101 | 0.078850 | 46.355 | < 2e-16*** |
| ageZ | -0.026814 | 0.073922 | -0.363 | 0.716819 |
| education | -1.081019 | 0.145802 | -7.414 | 1.47e-13*** |
| gender | -0.098495 | 0.169512 | -0.581 | 0.561240 |
| lonelinessZ | 1.841888 | 0.076447 | 24.094 | < 2e-16*** |
| covidInfectZ | 0.253933 | 0.069376 | 3.660 | 0.000255*** |
| Residual standard error: 4.545 on 4238 degrees of freedom  (231 observations deleted due to missingness)  Multiple R-squared: 0.5388, Adjusted R-squared: 0.5373  F-statistic: 353.6 on 14 and 4238 DF, p-value: < 2.2e-16 | | | | |

## Baseline Analyses: Effect Sizes

We calculated effect sizes of prosocial behavior subtypes (PB) on psychiatric symptoms based on the baseline regression models included in the main analyses. Effect sizes were estimated from the *t*-statistics of each predictor by first deriving the partial correlation coefficient (*r*), which reflects the unique association between each PB subtype and the outcome while controlling for the other predictors. Confidence intervals for *r* were obtained using Fisher’s *z* transformation. To facilitate interpretation, *r* values were converted to Cohen’s *d* equivalents.

Effect sizes were interpreted using conventional benchmarks ^14^

- *r* < 0.10 (≈ *d* < 0.20) – Very small effect
- 0.10 ≤ *r* < 0.30 (≈ *d* < 0.50) – Small effect
- 0.30 ≤ *r* < 0.50 (≈ *d* < 0.80) – Moderate effect
- *r* ≥ 0.50 (≈ *d* ≥ 0.80) – Large effect

Next, to evaluate whether these effects were clinically meaningful, we compared them to effect sizes from other relevant and partly independent predictors: anxiety and depression symptoms (in the substance use models), alcohol and substance use (in the anxiety and depression models).

**Note on effect size calculation.**
In a previous version of this manuscript (available as a preprint), effect sizes were reported as unique adjusted R² values for each predictor, reflecting the proportion of variance in the outcome explained above and beyond the other predictors. In the present version, we instead report partial correlations (r) and their Cohen’s d equivalents, derived from the t-statistics of the regression coefficients. Partial correlations are more standard effect size indices in regression contexts, directly quantify the unique strength of association between each predictor and the outcome, and allow for conversion into widely interpretable effect size metrics (Cohen’s d). Both approaches capture the unique contribution of predictors, but they are expressed on different scales (variance explained vs. association strength) and are therefore not directly comparable in magnitude. Nonetheless, the relative pattern of results across predictors remains consistent between the two approaches.

| **Supplementary Table S13.** Baseline Alcohol use and PB: Effect sizes | | | | | |
| --- | --- | --- | --- | --- | --- |
| **Predictor** | **t** | **r** | **r 95% CI** | **d** | **d 95% CI** |
| ptmAltruismZ | -3.238 | 0.050 | [0.02, 0.08] | 0.099 | [0.039, 0.16] |
| ptmAnynomousZ | -2.378 | 0.036 | [0.006, 0.066] | 0.073 | [0.013, 0.133] |
| ptmCompliantZ | 1.070 | 0.016 | [-0.014, 0.046] | 0.033 | [-0.027, 0.093] |
| ptmDireZ | 1.647 | 0.025 | [-0.005, 0.055] | 0.051 | [-0.01, 0.111] |
| ptmEmotionZ | 1.069 | 0.016 | [-0.014, 0.046] | 0.033 | [-0.027, 0.093] |
| ptmPublicZ | 1.056 | 0.016 | [-0.014, 0.046] | 0.032 | [-0.028, 0.093] |
| ageZ | -5.199 | 0.080 | [0.05, 0.109] | 0.160 | [0.099, 0.22] |
| education | 0.986 | 0.015 | [-0.015, 0.045] | 0.030 | [-0.03, 0.091] |
| gender | -9.117 | 0.139 | [0.109, 0.168] | 0.280 | [0.219, 0.341] |
| Note. t = test statistic; r = partial correlation coefficient; d = Cohen’s d equivalent effect size.  Confidence intervals (95% CI) are based on Fisher’s z transformation for r and back-transformed for d. | | | | | |

| **Supplementary Table S14.** Baseline Alcohol use and PB including clinical co-variates: Effect sizes | | | | | |
| --- | --- | --- | --- | --- | --- |
| **Predictor** | **t** | **r** | **r (95% CI)** | **d** | **d (95% CI)** |
| ptmAltruismZ | -3.258 | 0.050 | [0.02, 0.08] | 0.100 | [0.04, 0.16] |
| ptmAnynomousZ | -2.373 | 0.036 | [0.006, 0.066] | 0.073 | [0.013, 0.133] |
| ptmCompliantZ | 0.975 | 0.015 | [-0.015, 0.045] | 0.030 | [-0.03, 0.09] |
| ptmDireZ | 1.668 | 0.026 | [-0.004, 0.056] | 0.051 | [-0.009, 0.111] |
| ptmEmotionZ | 1.297 | 0.020 | [-0.01, 0.05] | 0.040 | [-0.02, 0.1] |
| ptmPublicZ | 1.113 | 0.017 | [-0.013, 0.047] | 0.034 | [-0.026, 0.094] |
| ageZ | -5.380 | 0.082 | [0.052, 0.112] | 0.165 | [0.105, 0.226] |
| education | 0.954 | 0.015 | [-0.015, 0.045] | 0.029 | [-0.031, 0.09] |
| gender | -8.887 | 0.135 | [0.106, 0.165] | 0.273 | [0.212, 0.334] |
| GAD7_t1Z | -2.228 | 0.034 | [0.004, 0.064] | 0.068 | [0.008, 0.129] |
| PHQ9_t1Z | 1.257 | 0.019 | [-0.011, 0.049] | 0.039 | [-0.022, 0.099] |
| Confidence intervals (95% CI) are based on Fisher’s z transformation for r and back-transformed for d.  **Abbreviations:** t = test statistic; r = partial correlation coefficient; d = Cohen’s d equivalent effect size; *GAD7* = Generalized Anxiety Disorder Scale-7; *PHQ-9* = Patient Health Questionnaire-9 | | | | | |

| **Supplementary Table S15.** Baseline Substance use and PB: Effect sizes | | | | | | | |
| --- | --- | --- | --- | --- | --- | --- | --- |
|  | **Predictor** | **t** | **r** | **r (95% CI)** | **d** | **d (95% CI)** |  |
|  | ptmAltruismZ | -1.321 | 0.020 | [-0.01, 0.05] | 0.041 | [-0.02, 0.101] |  |
|  | ptmAnynomousZ | -0.082 | 0.001 | [-0.029, 0.031] | 0.003 | [-0.058, 0.063] |  |
|  | ptmCompliantZ | -1.616 | 0.025 | [-0.005, 0.055] | 0.050 | [-0.011, 0.11] |  |
|  | ptmDireZ | 1.065 | 0.016 | [-0.014, 0.046] | 0.033 | [-0.027, 0.093] |  |
|  | ptmEmotionZ | 3.343 | 0.051 | [0.021, 0.081] | 0.103 | [0.042, 0.163] |  |
|  | ptmPublicZ | 2.108 | 0.032 | [0.002, 0.062] | 0.065 | [0.005, 0.125] |  |
|  | ageZ | -3.671 | 0.056 | [0.026, 0.086] | 0.113 | [0.052, 0.173] |  |
|  | education | -5.964 | 0.091 | [0.061, 0.121] | 0.183 | [0.123, 0.244] |  |
|  | gender | -9.519 | 0.145 | [0.115, 0.174] | 0.292 | [0.232, 0.353] |  |
|  | Confidence intervals (95% CI) are based on Fisher’s z transformation for r and back-transformed for d.  **Abbreviations:** t = test statistic; r = partial correlation coefficient; d = Cohen’s d equivalent effect size. | | | | | |  |

| **Supplementary Table S16.** Baseline Substance use and PB, including clinical co-variates: Effect sizes | | | | | |
| --- | --- | --- | --- | --- | --- |
| **Predictor** | **t** | **r** | **r (95% CI)** | **d** | **d (95% CI)** |
| ptmAltruismZ | -1.227 | 0.019 | [-0.011, 0.049] | 0.038 | [-0.023, 0.098] |
| ptmAnynomousZ | -0.698 | 0.011 | [-0.019, 0.041] | 0.021 | [-0.039, 0.082] |
| ptmCompliantZ | -1.621 | 0.025 | [-0.005, 0.055] | 0.050 | [-0.01, 0.11] |
| ptmDireZ | 1.007 | 0.015 | [-0.015, 0.046] | 0.031 | [-0.029, 0.091] |
| ptmEmotionZ | 2.708 | 0.042 | [0.011, 0.072] | 0.083 | [0.023, 0.143] |
| ptmPublicZ | 1.890 | 0.029 | [-0.001, 0.059] | 0.058 | [-0.002, 0.118] |
| ageZ | -3.189 | 0.049 | [0.019, 0.079] | 0.098 | [0.038, 0.158] |
| education | -4.611 | 0.071 | [0.041, 0.101] | 0.142 | [0.081, 0.202] |
| gender | -9.744 | 0.148 | [0.118, 0.177] | 0.299 | [0.239, 0.36] |
| GAD7_t1Z | 0.837 | 0.013 | [-0.017, 0.043] | 0.026 | [-0.035, 0.086] |
| PHQ9_t1Z | 5.268 | 0.081 | [0.051, 0.11] | 0.162 | [0.101, 0.222] |
| Confidence intervals (95% CI) are based on Fisher’s z transformation for r and back-transformed for d.  **Abbreviations:** t = test statistic; r = partial correlation coefficient; d = Cohen’s d equivalent effect size; *GAD7* = Generalized Anxiety Disorder Scale-7; *PHQ-9* = Patient Health Questionnaire-9 | | | | | |

| **Supplementary Table S17.** Baseline Anxiety and PB: Effect sizes | | | | | | |
| --- | --- | --- | --- | --- | --- | --- |
| **Predictor** | **t** | **r** | **r (95% CI)** | **d** | **d (95% CI)** |  |
| ptmAltruismZ | -0.886 | 0.014 | [-0.016, 0.044] | 0.027 | [-0.033, 0.087] |  |
| ptmAnynomousZ | 2.915 | 0.045 | [0.015, 0.075] | 0.089 | [0.029, 0.15] |  |
| ptmCompliantZ | -1.925 | 0.030 | [-0.001, 0.06] | 0.059 | [-0.001, 0.119] |  |
| ptmDireZ | 0.744 | 0.011 | [-0.019, 0.041] | 0.023 | [-0.037, 0.083] |  |
| ptmEmotionZ | 7.827 | 0.119 | [0.09, 0.149] | 0.240 | [0.18, 0.301] |  |
| ptmPublicZ | 2.256 | 0.035 | [0.005, 0.065] | 0.069 | [0.009, 0.13] |  |
| ageZ | -6.521 | 0.100 | [0.07, 0.129] | 0.200 | [0.14, 0.261] |  |
| education | -6.752 | 0.103 | [0.073, 0.133] | 0.207 | [0.147, 0.268] |  |
| gender | 4.954 | 0.076 | [0.046, 0.106] | 0.152 | [0.092, 0.213] |  |
| **Abbreviations:** t = test statistic; r = partial correlation coefficient; d = Cohen’s d equivalent effect size. Confidence intervals (95% CI) are based on Fisher’s z transformation for r and back-transformed for d. | | | | | |  |

| **Supplementary Table S18.** Baseline Anxiety and PB including clinical co-variates: Effect sizes | | | | | |
| --- | --- | --- | --- | --- | --- |
| **Predictor** | **t** | **r** | **r (95% CI)** | **d** | **d (95% CI)** |
| ptmAltruismZ | -0.918 | 0.014 | [-0.016, 0.044] | 0.028 | [-0.032, 0.088] |
| ptmAnynomousZ | 2.818 | 0.043 | [0.013, 0.073] | 0.087 | [0.026, 0.147] |
| ptmCompliantZ | -1.721 | 0.026 | [-0.004, 0.056] | 0.053 | [-0.007, 0.113] |
| ptmDireZ | 0.723 | 0.011 | [-0.019, 0.041] | 0.022 | [-0.038, 0.082] |
| ptmEmotionZ | 7.579 | 0.116 | [0.086, 0.145] | 0.233 | [0.172, 0.293] |
| ptmPublicZ | 2.110 | 0.032 | [0.002, 0.062] | 0.065 | [0.005, 0.125] |
| ageZ | -6.422 | 0.098 | [0.068, 0.128] | 0.197 | [0.137, 0.258] |
| education | -6.120 | 0.094 | [0.064, 0.123] | 0.188 | [0.128, 0.249] |
| gender | 5.374 | 0.082 | [0.052, 0.112] | 0.165 | [0.105, 0.226] |
| AUDIT_t1Z | -3.138 | 0.048 | [0.018, 0.078] | 0.096 | [0.036, 0.157] |
| DUDIT_t1Z | 6.262 | 0.096 | [0.066, 0.125] | 0.192 | [0.132, 0.253] |
| **Abbreviations:** t = test statistic; r = partial correlation coefficient; d = Cohen’s d equivalent effect size; *AUDIT* = Alcohol Use Disorder Identification Test; *DUDIT* = Drug Use Disorder Identification Test  Confidence intervals (95% CI) are based on Fisher’s z transformation for r and back-transformed for d. | | | | | |

| **Supplementary Table S19.** Baseline Depression and PB: Effect sizes | | | | | | |
| --- | --- | --- | --- | --- | --- | --- |
| **Predictor** | **t** | **r** | **r (95% CI)** | **d** | **d (95% CI)** |  |
| ptmAltruismZ | -0.820 | 0.013 | [-0.018, 0.043] | 0.025 | [-0.035, 0.085] |  |
| ptmAnynomousZ | 5.277 | 0.081 | [0.051, 0.111] | 0.162 | [0.102, 0.222] |  |
| ptmCompliantZ | 0.268 | 0.004 | [-0.026, 0.034] | 0.008 | [-0.052, 0.068] |  |
| ptmDireZ | 0.493 | 0.008 | [-0.023, 0.038] | 0.015 | [-0.045, 0.075] |  |
| ptmEmotionZ | 4.681 | 0.072 | [0.042, 0.102] | 0.144 | [0.083, 0.204] |  |
| ptmPublicZ | 1.795 | 0.028 | [-0.003, 0.058] | 0.055 | [-0.005, 0.115] |  |
| ageZ | -3.525 | 0.054 | [0.024, 0.084] | 0.108 | [0.048, 0.169] |  |
| education | -11.236 | 0.170 | [0.141, 0.199] | 0.345 | [0.284, 0.406] |  |
| gender | 1.052 | 0.016 | [-0.014, 0.046] | 0.032 | [-0.028, 0.093] |  |
| **Abbreviations:** t = test statistic; r = partial correlation coefficient; d = Cohen’s d equivalent effect size. Confidence intervals (95% CI) are based on Fisher’s z transformation for r and back-transformed for d. | | | | | |  |

| **Supplementary Table S20.** Baseline Depression and PB including clinical co-variates: Effect sizes | | | | | |
| --- | --- | --- | --- | --- | --- |
| **Predictor** | **t** | **r** | **r (95% CI)** | **d** | **d (95% CI)** |
| ptmAltruismZ | -0.758 | 0.012 | [-0.018, 0.042] | 0.023 | [-0.037, 0.084] |
| ptmAnynomousZ | 5.249 | 0.080 | [0.05, 0.11] | 0.161 | [0.101, 0.222] |
| ptmCompliantZ | 0.505 | 0.008 | [-0.022, 0.038] | 0.016 | [-0.045, 0.076] |
| ptmDireZ | 0.413 | 0.006 | [-0.024, 0.036] | 0.013 | [-0.048, 0.073] |
| ptmEmotionZ | 4.323 | 0.066 | [0.036, 0.096] | 0.133 | [0.072, 0.193] |
| ptmPublicZ | 1.575 | 0.024 | [-0.006, 0.054] | 0.048 | [-0.012, 0.109] |
| ageZ | -3.236 | 0.050 | [0.02, 0.08] | 0.099 | [0.039, 0.16] |
| education | -10.489 | 0.159 | [0.13, 0.188] | 0.322 | [0.261, 0.383] |
| gender | 1.942 | 0.030 | [0, 0.06] | 0.060 | [-0.001, 0.12] |
| AUDIT_t1Z | -1.951 | 0.030 | [0, 0.06] | 0.060 | [0, 0.12] |
| DUDIT_t1Z | 7.989 | 0.122 | [0.092, 0.151] | 0.245 | [0.185, 0.306] |
| **Abbreviations:** t = test statistic; r = partial correlation coefficient; d = Cohen’s d equivalent effect size; *AUDIT* = Alcohol Use Disorder Identification Test; *DUDIT* = Drug Use Disorder Identification Test  Confidence intervals (95% CI) are based on Fisher’s z transformation for r and back-transformed for d. | | | | | |

## Baseline Analyses: Exploratory Models (ADHD; OCD; PTSD; Gambling)

An additional set of psychiatric symptom measures were assessed at baseline only for exploratory purposes: Attention Deficit Hyperactivity Disorder (using The Barkley Adult ADHD Rating Scale-IV^8^), Obsessive Compulsive Disorder (Obsessive-Compulsive Inventory-Revised ^9^), Posttraumatic Stress Disorder (The Posttraumatic Stress Disorder Checklist ^10^), and Problem Gambling (Problem Gambling Severity Index ^11^), Similarly, as for our main baseline models, we adjusted for demographics and the remaining psychiatric symptom scales.

| **Supplementary Table S21.** Baseline ADHD (BAARS) model | | | | |
| --- | --- | --- | --- | --- |
|  | Estimate | Std. Error | t value | Pr(>\|t\|) |
| (Intercept) | 18.4086 | 0.9142 | 20.136 | < 2e-16*** |
| ptmAltruismZ | -0.2622 | 0.2099 | -1.249 | 0.211718 |
| ptmAnynomousZ | 0.7913 | 0.1841 | 4.299 | 1.75e-05*** |
| ptmCompliantZ | -0.7846 | 0.2272 | -3.453 | 0.000559*** |
| ptmDireZ | 0.8619 | 0.2507 | 3.438 | 0.000591*** |
| ptmEmotionZ | 0.1988 | 0.2269 | 0.876 | 0.380900 |
| ptmPublicZ | 0.7377 | 0.2091 | 3.528 | 0.000424*** |
| AUDITZ | 0.6291 | 0.1797 | 3.500 | 0.000470*** |
| DUDITZ | 0.9157 | 0.1819 | 5.033 | 5.02e-07*** |
| GAD7Z | 3.7746 | 0.2385 | 15.826 | < 2e-16*** |
| PHQ9Z | 4.1561 | 0.2374 | 17.507 | < 2e-16*** |
| ageZ | -0.4840 | 0.1826 | -2.651 | 0.008057** |
| education | -0.7480 | 0.3630 | -2.061 | 0.039381* |
| gender | 0.3932 | 0.4175 | 0.942 | 0.346304 |
| Residual standard error: 11.25 on 4239 degrees of freedom  (231 observations deleted due to missingness)  Multiple R-squared: 0.336, Adjusted R-squared: 0.3339  F-statistic: 165 on 13 and 4239 DF, p-value: < 2.2e-16 | | | | |

| **Supplementary Table S22.** Baseline OCD model | | | | |
| --- | --- | --- | --- | --- |
|  | Estimate | Std. Error | t value | Pr(>\|t\|) |
| (Intercept) | 12.2445 | 1.0243 | 11.954 | < 2e-16*** |
| ptmAltruismZ | -0.9097 | 0.2352 | -3.868 | 0.000111*** |
| ptmAnynomousZ | 0.5500 | 0.2062 | 2.667 | 0.007685** |
| ptmCompliantZ | -0.2364 | 0.2545 | -0.929 | 0.353034 |
| ptmDireZ | -0.3970 | 0.2809 | -1.414 | 0.157569 |
| ptmEmotionZ | 0.5586 | 0.2542 | 2.197 | 0.028055* |
| ptmPublicZ | 0.6368 | 0.2343 | 2.718 | 0.006595** |
| AUDITZ | 0.4021 | 0.2014 | 1.997 | 0.045915* |
| DUDITZ | 0.7276 | 0.2038 | 3.569 | 0.000362*** |
| GAD7Z | 4.1267 | 0.2672 | 15.443 | < 2e-16*** |
| PHQ9Z | 1.1430 | 0.2660 | 4.297 | 1.77e-05*** |
| ageZ | -1.0388 | 0.2046 | -5.078 | 3.97e-07*** |
| education | -2.3080 | 0.4066 | -5.676 | 1.47e-08*** |
| gender | 0.3835 | 0.4677 | 0.820 | 0.412236 |
| Residual standard error: 12.61 on 4239 degrees of freedom  (231 observations deleted due to missingness)  Multiple R-squared: 0.1941, Adjusted R-squared: 0.1916  F-statistic: 78.52 on 13 and 4239 DF, p-value: < 2.2e-16 | | | | |

| **Supplementary Table S23.** Baseline PTSD (PCL-5) model | | | | |
| --- | --- | --- | --- | --- |
|  | Estimate | Std. Error | t value | Pr(>\|t\|) |
| (Intercept) | 19.07277 | 1.55693 | 12.250 | < 2e-16*** |
| ptmAltruismZ | -0.03770 | 0.35746 | -0.105 | 0.91601 |
| ptmAnynomousZ | 1.28381 | 0.31346 | 4.096 | 4.29e-05*** |
| ptmCompliantZ | -1.02857 | 0.38689 | -2.659 | 0.00788** |
| ptmDireZ | 2.63843 | 0.42689 | 6.181 | 6.98e-10*** |
| ptmEmotionZ | 0.98366 | 0.38641 | 2.546 | 0.01094* |
| ptmPublicZ | 0.08221 | 0.35613 | 0.231 | 0.81745 |
| AUDITZ | -0.32181 | 0.30608 | -1.051 | 0.29314 |
| DUDITZ | 2.35714 | 0.30982 | 7.608 | 3.41e-14*** |
| GAD7Z | 3.84197 | 0.40621 | 9.458 | < 2e-16*** |
| PHQ9Z | 6.60629 | 0.40426 | 16.342 | < 2e-16*** |
| ageZ | 2.14697 | 0.31103 | 6.903 | 5.86e-12*** |
| education | -1.89136 | 0.61810 | -3.060 | 0.00223** |
| gender | 3.30673 | 0.71095 | 4.651 | 3.40e-06*** |
| Residual standard error: 19.16 on 4238 degrees of freedom  (232 observations deleted due to missingness)  Multiple R-squared: 0.2629, Adjusted R-squared: 0.2607  F-statistic: 116.3 on 13 and 4238 DF, p-value: < 2.2e-16 | | | | |

| **Supplementary Table S24.** Baseline Problem Gambling model | | | | |
| --- | --- | --- | --- | --- |
|  | Estimate | Std. Error | t value | Pr(>\|t\|) |
| (Intercept) | 1.045976 | 0.154174 | 6.784 | 1.33e-11*** |
| ptmAltruismZ | -0.036203 | 0.035399 | -1.023 | 0.306504 |
| ptmAnynomousZ | -0.002667 | 0.031042 | -0.086 | 0.931544 |
| ptmCompliantZ | 0.010736 | 0.038313 | 0.280 | 0.779316 |
| ptmDireZ | 0.106924 | 0.042274 | 2.529 | 0.011465* |
| ptmEmotionZ | -0.052763 | 0.038263 | -1.379 | 0.167983 |
| ptmPublicZ | 0.074167 | 0.035267 | 2.103 | 0.035522* |
| AUDITZ | 0.089621 | 0.030310 | 2.957 | 0.003126** |
| DUDITZ | 0.047167 | 0.030681 | 1.537 | 0.124289 |
| GAD7Z | 0.162062 | 0.040222 | 4.029 | 5.69e-05*** |
| PHQ9Z | 0.032413 | 0.040034 | 0.810 | 0.418187 |
| ageZ | 0.135560 | 0.030790 | 4.403 | 1.10e-05*** |
| education | -0.217270 | 0.061209 | -3.550 | 0.000390*** |
| gender | -0.250582 | 0.070400 | -3.559 | 0.000376*** |
| Residual standard error: 1.898 on 4239 degrees of freedom  (231 observations deleted due to missingness)  Multiple R-squared: 0.03051, Adjusted R-squared: 0.02753  F-statistic: 10.26 on 13 and 4239 DF, p-value: < 2.2e-16 | | | | |

## Longitudinal Analyses: Main Models

One latent class mixed model was estimated per psychiatric measure, differing in the number of latent classes included. Classes were added to the model, based on that the percentage of participants per class remained at least 10% of the sample. In other words, models in which one class included less than 10% of participants were not considered, and no more classes were added.

The final model selection (in bold) was based on the Bayesian Information Criterion (BIC).

Alcohol use models:

| **Supplementary Table S25.** Alcohol use model selection | | | | | | | |
| --- | --- | --- | --- | --- | --- | --- | --- |
|  | G | loglik | npm | BIC | %class1 | %class2 | %class3 |
| gmm1 | 1 | -20483.31 | 8 | 41031.37 | 100.00000 |  |  |
| **gmm2** | **2** | **-19994.43** | **16** | **40118.35** | **31.94742** | **68.052583** |  |
| gmm3 | 3 | -19701.34 | 24 | 39596.91 | 32.19199 | 3.668603 | 64.13941 |

| **Supplementary Table S26.** Substance use model selection | | | | | | | |
| --- | --- | --- | --- | --- | --- | --- | --- |
|  | G | loglik | npm | BIC | %class1 | %class2 | %class3 |
| gmm1 | 1 | -18195.67 | 8 | 36455.44 | 100.00000 |  |  |
| **gmm2** | **2** | **-15341.01** | **16** | **30810.19** | **86.82814** | **13.17186** |  |
| gmm3 | 3 | -14417.97 | 24 | 29028.21 | 84.83743 | 9.52223 | 5.640345 |

| **Supplementary Table S27.** Anxiety model selection | | | | | | | |
| --- | --- | --- | --- | --- | --- | --- | --- |
|  | G | loglik | npm | BIC | %class1 | %class2 | %class3 |
| gmm1 | 1 | -65908.74 | 15 | 131939.8 | 100.00000 |  |  |
| gmm2 | 2 | -65525.46 | 30 | 131295.5 | 10.39747 | 89.60253 |  |
| **gmm3** | **3** | **-65310.79** | **45** | **130988.5** | **49.71198** | **11.29032** | **38.9977** |

| **Supplementary Table S28.** Depression model selection | | | | | | | |
| --- | --- | --- | --- | --- | --- | --- | --- |
|  | G | loglik | npm | BIC | %class1 | %class2 | %class3 |
| gmm1 | 1 | -69366.54 | 15 | 138855.4 | 100.00000 |  |  |
| **gmm2** | **2** | **-68967.46** | **30** | **138179.6** | **87.44253** | **12.55747** |  |
| gmm3 | 3 | -68773.40 | 45 | 137913.8 | 10.22989 | 85.11494 | 4.655172 |

Following classifying individuals into different classes for each psychiatric domain, we applied a logistic regression model with the classification variable as a dependent variable and adding the six prosocial subtypes, demographic variables and the other psychiatric symptom classes as independent variables.

For the Alcohol, Substance use, and Depression models, we used a binary logistic regression model, given that these models had two latent classes (reference level = class 2/low symptom class). For the anxiety model, we used a multinomial logistic regression model, given that it had three latent classes (reference level = class 3/low symptom class).

*Generic logistic regression model specification:*

*classVariable1 ~ ptmAltruismZ + ptmAnynomousZ + ptmCompliantZ+ ptmDireZ + ptmEmotionZ+ ptmPublicZ + classVariable2Z + classVariable3Z + classVariable4Z + ageZ + education + gender*

| **Supplementary Table S29.** Longitudinal Alcohol use-model | | | |
| --- | --- | --- | --- |
| **Predictor** | **OR** | **95% CI** | **p** |
| ptmAltruismZ | 0.96 | 0.86–1.07 | .433 |
| ptmAnynomousZ | 0.89 | 0.82–0.98 | .013 |
| ptmCompliantZ | 1.05 | 0.94–1.17 | .386 |
| ptmDireZ | 0.94 | 0.83–1.06 | .322 |
| ptmEmotionZ | 1.09 | 0.98–1.22 | .131 |
| ptmPublicZ | 0.99 | 0.89–1.1 | .837 |
| class_SUDZ | 0.65 | 0.58–0.72 | <.001 |
| class_GADZ | 1.22 | 1.12–1.33 | <.001 |
| class_PHQZ | 1.03 | 0.95–1.12 | .420 |
| ageZ | 0.83 | 0.76–0.9 | <.001 |
| education | 1.63 | 1.37–1.94 | <.001 |
| gender | 1.02 | 0.82–1.25 | .889 |
| Note. For binary logistic regression, each row compares the the reference class (= Class 2/Low symptom class). OR = odds ratio. CI = confidence interval.  N = 2663; AIC = 3196.1. | | | |

| **Supplementary Table S30.** Longitudinal Substance use-model | | | |
| --- | --- | --- | --- |
| **Predictor** | **OR** | **95% CI** | **p** |
| ptmAltruismZ | 1.00 | 0.88–1.15 | .963 |
| ptmAnynomousZ | 1.01 | 0.89–1.14 | .925 |
| ptmCompliantZ | 0.93 | 0.8–1.09 | .387 |
| ptmDireZ | 1.06 | 0.89–1.26 | .500 |
| ptmEmotionZ | 1.06 | 0.91–1.23 | .458 |
| ptmPublicZ | 1.15 | 1.01–1.31 | .036 |
| class_AUDZ | 0.55 | 0.47–0.63 | <.001 |
| class_GADZ | 0.78 | 0.69–0.88 | <.001 |
| class_PHQZ | 1.00 | 0.89–1.11 | .969 |
| ageZ | 1.00 | 0.89–1.13 | .978 |
| education | 0.63 | 0.5–0.8 | <.001 |
| gender | 0.52 | 0.4–0.68 | <.001 |
| Note. For binary logistic regression, each row compares the the reference class (= Class 2/Low symptom class). OR = odds ratio. CI = confidence interval.  N = 2663; AIC = 1960.6. | | | |

| **Supplementary Table S31.** Longitudinal Anxiety-model | | | | |
| --- | --- | --- | --- | --- |
| **Class** | **Predictor** | **OR** | **95% CI** | **p** |
| 1 | ptmAltruismZ | 1.00 | 0.9–1.12 | .987 |
| 1 | ptmAnynomousZ | 1.09 | 0.99–1.19 | .070 |
| 1 | ptmCompliantZ | 0.94 | 0.84–1.05 | .271 |
| 1 | ptmDireZ | 1.08 | 0.96–1.23 | .205 |
| 1 | ptmEmotionZ | 1.25 | 1.12–1.4 | <.001 |
| 1 | ptmPublicZ | 1.10 | 0.99–1.22 | .089 |
| 1 | class_AUDZ | 1.22 | 1.12–1.33 | <.001 |
| 1 | class_SUDZ | 0.84 | 0.77–0.92 | <.001 |
| 1 | class_PHQZ | 0.74 | 0.67–0.82 | <.001 |
| 1 | ageZ | 0.79 | 0.73–0.87 | <.001 |
| 1 | education | 0.67 | 0.56–0.81 | <.001 |
| 1 | gender | 1.29 | 1.04–1.59 | .019 |
| 2 | ptmAltruismZ | 1.04 | 0.88–1.23 | .629 |
| 2 | ptmAnynomousZ | 1.05 | 0.91–1.2 | .515 |
| 2 | ptmCompliantZ | 1.05 | 0.88–1.24 | .589 |
| 2 | ptmDireZ | 0.87 | 0.73–1.05 | .150 |
| 2 | ptmEmotionZ | 1.22 | 1.04–1.45 | .017 |
| 2 | ptmPublicZ | 1.19 | 1.01–1.39 | .037 |
| 2 | class_AUDZ | 1.04 | 0.91–1.19 | .575 |
| 2 | class_SUDZ | 0.89 | 0.78–1.02 | .090 |
| 2 | class_PHQZ | 1.85 | 1.69–2.03 | <.001 |
| 2 | ageZ | 0.84 | 0.74–0.96 | .008 |
| 2 | education | 0.83 | 0.63–1.08 | .166 |
| 2 | gender | 1.50 | 1.08–2.09 | .016 |
| Note. For multinomial logistic regression, each row compares the indicated class with the reference class (= Class 3/Low symptom class). OR = odds ratio. CI = confidence interval.  N = 2663; AIC = 4831.3. | | | | |

| **Supplementary Table S32.** Longitudinal Depression-model | | | |
| --- | --- | --- | --- |
| **Predictor** | **OR** | **95% CI** | **p** |
| ptmAltruismZ | 1.02 | 0.89–1.17 | .746 |
| ptmAnynomousZ | 0.99 | 0.88–1.11 | .840 |
| ptmCompliantZ | 0.95 | 0.82–1.09 | .450 |
| ptmDireZ | 0.93 | 0.79–1.08 | .330 |
| ptmEmotionZ | 1.07 | 0.93–1.23 | .317 |
| ptmPublicZ | 1.09 | 0.94–1.25 | .259 |
| class_AUDZ | 1.04 | 0.93–1.17 | .447 |
| class_SUDZ | 1.00 | 0.89–1.11 | .969 |
| class_GADZ | 0.72 | 0.65–0.81 | <.001 |
| ageZ | 1.17 | 1.04–1.31 | .008 |
| education | 0.94 | 0.75–1.19 | .629 |
| gender | 0.93 | 0.7–1.22 | .598 |
| Note. For binary logistic regression, each row compares the the reference class (= Class 2/Symptom-improvement class). OR = odds ratio. CI = confidence interval.  N = 2663; AIC = 2223.8. | | | |

## Longitudinal Analyses: Extended Models

**Controlling for COVID-19-related variables: infection and subjective loneliness.**

| **Supplementary Table S33.** Longitudinal Alcohol use COVID-model | | | |
| --- | --- | --- | --- |
| **Predictor** | **OR** | **95% CI** | **p** |
| ptmAltruismZ | 0.96 | 0.86–1.07 | .440 |
| ptmAnynomousZ | 0.90 | 0.82–0.98 | .020 |
| ptmCompliantZ | 1.05 | 0.94–1.18 | .354 |
| ptmDireZ | 0.93 | 0.82–1.05 | .260 |
| ptmEmotionZ | 1.10 | 0.98–1.23 | .098 |
| ptmPublicZ | 0.99 | 0.9–1.11 | .920 |
| class_SUDZ | 0.65 | 0.58–0.72 | <.001 |
| class_GADZ | 1.18 | 1.08–1.29 | <.001 |
| class_PHQZ | 1.04 | 0.96–1.13 | .322 |
| ageZ | 0.83 | 0.77–0.91 | <.001 |
| education | 1.59 | 1.34–1.9 | <.001 |
| gender | 1.00 | 0.81–1.23 | .989 |
| lonelinessZ | 0.87 | 0.8–0.96 | .003 |
| covidInfectZ | 0.96 | 0.88–1.05 | .339 |
| Note. For binary logistic regression, each row compares the the reference class (= Class 2/Low symptom class). OR = odds ratio. CI = confidence interval.  N = 2663; AIC = 3190.6. | | | |

| **Supplementary Table S34.** Longitudinal Substance use COVID-model | | | |
| --- | --- | --- | --- |
| **Predictor** | **OR** | **95% CI** | **p** |
| ptmAltruismZ | 1.00 | 0.88–1.16 | .957 |
| ptmAnynomousZ | 1.00 | 0.89–1.14 | .945 |
| ptmCompliantZ | 0.93 | 0.8–1.09 | .384 |
| ptmDireZ | 1.06 | 0.9–1.26 | .484 |
| ptmEmotionZ | 1.06 | 0.91–1.23 | .472 |
| ptmPublicZ | 1.15 | 1.01–1.31 | .038 |
| class_AUDZ | 0.55 | 0.47–0.63 | <.001 |
| class_GADZ | 0.79 | 0.69–0.9 | <.001 |
| class_PHQZ | 1.00 | 0.89–1.11 | .946 |
| ageZ | 1.00 | 0.89–1.12 | .997 |
| education | 0.63 | 0.5–0.8 | <.001 |
| gender | 0.52 | 0.4–0.68 | <.001 |
| lonelinessZ | 1.03 | 0.91–1.17 | .608 |
| covidInfectZ | 1.00 | 0.88–1.12 | .977 |
| Note. For binary logistic regression, each row compares the the reference class (= Class 2/Low symptom class). OR = odds ratio. CI = confidence interval.  N = 2663; AIC = 1964.4. | | | |

| **Supplementary Table S35.** Longitudinal Anxiety COVID-model | | | | |
| --- | --- | --- | --- | --- |
| **Class** | **Predictor** | **OR** | **95% CI** | **p** |
| 1 | ptmAltruismZ | 1.01 | 0.9–1.13 | .897 |
| 1 | ptmAnynomousZ | 1.06 | 0.96–1.17 | .220 |
| 1 | ptmCompliantZ | 0.92 | 0.82–1.04 | .186 |
| 1 | ptmDireZ | 1.14 | 1.01–1.3 | .040 |
| 1 | ptmEmotionZ | 1.20 | 1.07–1.35 | .002 |
| 1 | ptmPublicZ | 1.08 | 0.97–1.21 | .157 |
| 1 | class_AUDZ | 1.18 | 1.08–1.29 | <.001 |
| 1 | class_SUDZ | 0.85 | 0.77–0.93 | <.001 |
| 1 | class_PHQZ | 0.73 | 0.66–0.81 | <.001 |
| 1 | ageZ | 0.77 | 0.71–0.85 | <.001 |
| 1 | education | 0.75 | 0.62–0.9 | .002 |
| 1 | gender | 1.36 | 1.09–1.69 | .006 |
| 1 | uclalsZ | 1.82 | 1.66–1.99 | <.001 |
| 1 | covidInfectZ | 1.00 | 0.91–1.09 | .941 |
| 2 | ptmAltruismZ | 1.03 | 0.87–1.22 | .713 |
| 2 | ptmAnynomousZ | 1.03 | 0.89–1.19 | .687 |
| 2 | ptmCompliantZ | 1.03 | 0.86–1.22 | .767 |
| 2 | ptmDireZ | 0.94 | 0.78–1.13 | .504 |
| 2 | ptmEmotionZ | 1.17 | 0.99–1.39 | .064 |
| 2 | ptmPublicZ | 1.16 | 0.98–1.36 | .077 |
| 2 | class_AUDZ | 1.01 | 0.88–1.16 | .922 |
| 2 | class_SUDZ | 0.90 | 0.78–1.03 | .110 |
| 2 | class_PHQZ | 1.82 | 1.65–2 | <.001 |
| 2 | ageZ | 0.82 | 0.72–0.93 | .003 |
| 2 | education | 0.90 | 0.68–1.18 | .439 |
| 2 | gender | 1.59 | 1.14–2.23 | .007 |
| 2 | uclalsZ | 1.77 | 1.53–2.03 | <.001 |
| 2 | covidInfectZ | 1.00 | 0.87–1.14 | .977 |
| Note. OR = odds ratio. CI = confidence interval. For multinomial logistic regression, each row compares the indicated class with the reference class (= Class 3/Low symptom class).  N = 2663; AIC = 4643.1. | | | | |

| **Supplementary Table S36.** Longitudinal Depression COVID-model | | | | |
| --- | --- | --- | --- | --- |
| **Predictor** | **OR** | **95% CI** | **p** |  |
| ptmAltruismZ | 1.02 | 0.89–1.17 | .760 |  |
| ptmAnynomousZ | 0.99 | 0.88–1.12 | .914 |  |
| ptmCompliantZ | 0.95 | 0.83–1.1 | .521 |  |
| ptmDireZ | 0.91 | 0.78–1.06 | .234 |  |
| ptmEmotionZ | 1.09 | 0.95–1.25 | .236 |  |
| ptmPublicZ | 1.09 | 0.95–1.26 | .221 |  |
| class_SUDZ | 1.06 | 0.94–1.19 | .343 |  |
| class_GADZ | 1.00 | 0.89–1.11 | .965 |  |
| class_PHQZ | 0.69 | 0.61–0.77 | <.001 |  |
| ageZ | 1.18 | 1.05–1.32 | .005 |  |
| education | 0.92 | 0.73–1.15 | .468 |  |
| gender | 0.91 | 0.69–1.19 | .486 |  |
| lonelinessZ | 0.83 | 0.74–0.93 | .001 |  |
| covidInfectZ | 0.94 | 0.85–1.05 | .276 |  |
| Note. For binary logistic regression, each row compares the the reference class (= Class 2/symptom-improvement class). OR = odds ratio. CI = confidence interval.  N = 2663; AIC = 2216.3. | | | |  |

Longitudinal Analyses: Trajectory Characteristics

| **Supplementary Table S37.** Associations between Participant Characteristics and Alcohol Use Trajectories | | | |
| --- | --- | --- | --- |
| **Alcohol use trajectories** | **High symptom profile**  **(Class 1, N = 2103)** | **Low symptom profile**  **(Class 2, N = 989)** | **Test statistic (T-test, *P*)*** |
| Age, *mean (SD)* | 36.20 (12.58) | 37.86 (12.36) | 0.001 |
| Gender, *n male (%)* | 1618 (76.9) | 761 (76.9) | 1.000 |
| Education, Undergraduate degree or higher, *n (%)* | 1266 (60.2) | 515 (52.1) | <0.001 |
| Lifetime diagnosis |  |  |  |
| Alcohol or substance use disorder, *n (%)* | 0.07 (0.26) | 0.10 (0.30) | 0.016 |
| Anxiety disorder | 1454 (69.1) | 729 (73.7) | 0.010 |
| Depressive disorder | 1638 (77.9) | 785 (79.4) | 0.374 |
| Current psychiatric treatment, *n (%)* |  |  |  |
| Psychotherapy | 499 (23.7) | 239 (24.2) | 0.825 |
| Pharmacotherapy | 1047 (49.8) | 513 (51.9) | 0.297 |
| Psychiatric symptom load at baseline, *mean (SD)* |  |  |  |
| AUDIT | 10.18 (4.72) | 2.60 (3.85) | <0.001 |
| DUDIT | 1.51 (4.37) | 0.80 (3.46) | <0.001 |
| GAD-7 | 11.64 (5.63) | 12.24 (5.81) | 0.006 |
| PHQ-9 | 14.72 (6.54) | 15.67 (6.87) | <0.001 |
| Prosocial behavior (PB), m*ean (SD)* |  |  |  |
| Altruistic PB | 23.10 (2.41) | 23.27 (2.57) | 0.067 |
| Anonymous PB | 12.91 (4.46) | 13.35 (4.85) | 0.012 |
| Compliant PB | 7.45 (1.91) | 7.37 (2.02) | 0.328 |
| Dire PB | 10.42 (2.88) | 10.39 (3.02) | 0.768 |
| Emotional PB | 11.72 (3.46) | 11.46 (3.55) | 0.052 |
| Public PB | 5.62 (2.32) | 5.54 (2.26) | 0.381 |
|  |  |  |  |
| * The results are based on multiple t-tests to illustrate general group differences between classes.  **Abbreviations:** AUDIT = Alcohol Use Disorder Identification Test; DUDIT = Drug Use Disorder Identificaiton Test;  GAD-7 = Generalized Anxiety Disorder-7; PB = Prosocial Behavior, PHQ-9 = Patient Health Questionnaire-9 | | | |

| **Supplementary Table S38.** Associations between Participant Characteristics and Substance Use Trajectories | | | |
| --- | --- | --- | --- |
| **Alcohol use trajectories** | **High symptom profile**  **(Class 1, N = 376)** | **Low symptom profile**  **(Class 2, N = 2464)** | **Test statistic (T-test, *P*)*** |
| Age, *mean (SD)* | 36.34 (13.12) | 37.20 (12.49) | 0.215 |
| Gender, *n male (%)* | 253 (67.3) | 1948 (79.1) | <0.001 |
| Education, Undergraduate degree or higher, *n (%)* | 187 (49.7) | 1481 (60.1) | <0.001 |
| Lifetime diagnosis |  |  |  |
| Alcohol or substance use disorder, *n (%)* | 0.21 (0.41) | 0.06 (0.24) | <0.001 |
| Anxiety disorder | 280 (74.5) | 1720 (69.8) | 0.074 |
| Depressive disorder | 313 (83.2) | 1926 (78.2) | 0.029 |
| Current psychiatric treatment, *n (%)* |  |  |  |
| Psychotherapy | 86 (22.9) | 603 (24.5) | 0.542 |
| Pharmacotherapy | 199 (52.9) | 1241 (50.4) | 0.385 |
| Psychiatric symptom load at baseline, *mean (SD)* |  |  |  |
| AUDIT | 11.22 (6.62) | 7.23 (5.30) | <0.001 |
| DUDIT | 7.08 (7.86) | 0.42 (2.01) | <0.001 |
| GAD-7 | 12.29 (5.59) | 11.80 (5.80) | 0.120 |
| PHQ-9 | 16.50 (6.50) | 14.89 (6.68) | <0.001 |
| Prosocial behavior (PB), m*ean (SD)* |  |  |  |
| Altruistic PB | 22.85 (2.51) | 23.26 (2.36) | 0.002 |
| Anonymous PB | 13.19 (4.47) | 13.08 (4.61) | 0.670 |
| Compliant PB | 7.44 (1.88) | 7.43 (1.98) | 0.889 |
| Dire PB | 10.52 (2.88) | 10.43 (2.92) | 0.555 |
| Emotional PB | 12.03 (3.56) | 11.58 (3.45) | 0.019 |
| Public PB | 6.05 (2.61) | 5.50 (2.21) | <0.001 |
|  |  |  |  |
| * The results are based on multiple t-tests to illustrate general group differences between classes.  **Abbreviations:** AUDIT = Alcohol Use Disorder Identification Test; DUDIT = Drug Use Disorder Identificaiton Test;  GAD-7 = Generalized Anxiety Disorder-7; PB = Prosocial Behavior, PHQ-9 = Patient Health Questionnaire-9 | | | |

| **Supplementary Table S39.** Associations between Participant Characteristics and Anxiety Trajectories | | | | |
| --- | --- | --- | --- | --- |
| **Anxiety trajectories** | **High symptom profile**  **(Class 1, N = 1622)** | **Improved-symptom profile**  **(Class 2, N = 374)** | **Low symptom profile**  **(Class 3, N = 1282)** | **Test statistic (ANOVA / Chi-square, *P*)*** |
| Age, *mean (SD)* | 35.24 (11.91) | 35.90 (12.50) | 38.57 (12.88) | <0.001 |
| Gender, *n male (%)* | 1270 (78.3) | 302 (80.7) | 941 (73.4) | 0.001 |
| Education, Undergraduate degree or higher, *n (%)* | 822 (50.7) | 222 (59.4) | 821 (64.0) | <0.001 |
| Lifetime diagnosis |  |  |  |  |
| Alcohol or substance use disorder, *n (%)* | 150 (9.2) | 26 (7.0) | 91 (7.1) | 0.073 |
| Anxiety disorder | 1269 (78.2) | 270 (72.2) | 776 (60.5) | <0.001 |
| Depressive disorder | 1332 (82.1) | 298 (79.7) | 942 (73.5) | <0.001 |
| Current psychiatric treatment, *n (%)* |  |  |  |  |
| Psychotherapy | 0.55 (0.50) | 0.56 (0.50) | 0.43 (0.50) | <0.001 |
| Pharmacotherapy | 0.28 (0.45) | 0.28 (0.45) | 0.18 (0.38) | <0.001 |
| Psychiatric symptom load at baseline, *mean (SD)* |  |  |  |  |
| AUDIT | 7.71 (6.13) | 7.89 (5.48) | 7.84 (5.35) | 0.762 |
| DUDIT | 1.77 (5.00) | 1.21 (3.99) | 0.80 (2.68) | <0.001 |
| GAD-7 | 14.62 (4.63) | 14.41 (4.54) | 7.73 (4.67) | <0.001 |
| PHQ-9 | 17.87 (5.85) | 16.76 (5.90) | 11.17 (5.90) | <0.001 |
| Prosocial behavior (PB), m*ean (SD)* |  |  |  |  |
| Altruistic PB | 23.07 (2.65) | 23.20 (2.21) | 23.25 (2.29) | 0.133 |
| Anonymous PB | 13.24 (4.76) | 13.10 (4.42) | 12.75 (4.48) | 0.018 |
| Compliant PB | 7.49 (1.98) | 7.51 (1.97) | 7.32 (1.90) | 0.038 |
| Dire PB | 10.61 (2.93) | 10.36 (2.89) | 10.18 (2.91) | <0.001 |
| Emotional PB | 12.10 (3.45) | 11.74 (3.39) | 11.11 (3.49) | <0.001 |
| Public PB | 5.67 (2.42) | 5.64 (2.37) | 5.50 (2.17) | 0.127 |
|  |  |  |  |  |
| * The results are based on multiple ANOVA's (continous variables) or ChiSqaure (binary variables) to illustrate general group differences between classes.  **Abbreviations:** AUDIT = Alcohol Use Disorder Identification Test; DUDIT = Drug Use Disorder Identificaiton Test;  GAD-7 = Generalized Anxiety Disorder-7; PB = Prosocial Behavior, PHQ-9 = Patient Health Questionnaire-9 | | | | |

| **Supplementary Table S40** Associations between Participant Characteristics and Depression Trajectories | | | | |
| --- | --- | --- | --- | --- |
| **Depression trajectories** | **High symptom profile**  **(Class 1, N = 2876)** | **Symptom-improvement profile**  **(Class 2, N = 410)** | | **Test statistic (T-test, *P*)*** |
| Age, *mean (SD)* | 36.66 (12.40) | 36.18 (12.86) | 0.460 | |
| Gender, *n male (%)* | 2197 (76.4) | 323 (78.8) | 0.313 | |
| Education, Undergraduate degree or higher, *n (%)* | 1613 (56.1) | 253 (61.7) | 0.036 | |
| Lifetime diagnosis |  |  |  | |
| Alcohol or substance use disorder, *n (%)* | 242 (8.4) | 28 (6.8) | 0.319 | |
| Anxiety disorder | 2044 (71.1) | 276 (67.3) | 0.133 | |
| Depressive disorder | 2256 (78.4) | 320 (78.0) | 0.907 | |
| Current psychiatric treatment, *n (%)* |  |  |  | |
| Psychotherapy | 0.50 (0.50) | 0.55 (0.50) | 0.043 | |
| Pharmacotherapy | 0.24 (0.43) | 0.23 (0.42) | 0.501 | |
| Psychiatric symptom load at baseline, *mean (SD)* |  |  |  | |
| AUDIT | 7.77 (5.78) | 7.99 (5.64) | 0.470 | |
| DUDIT | 1.38 (4.24) | 0.94 (3.39) | 0.044 | |
| GAD-7 | 11.80 (5.77) | 12.67 (5.25) | 0.004 | |
| PHQ-9 | 14.97 (6.76) | 16.24 (5.99) | <0.001 | |
| Prosocial behavior (PB), m*ean (SD)* |  |  |  | |
| Altruistic PB | 23.13 (2.52) | 23.30 (2.12) | 0.190 | |
| Anonymous PB | 13.00 (4.63) | 13.20 (4.55) | 0.431 | |
| Compliant PB | 7.41 (1.95) | 7.54 (1.94) | 0.189 | |
| Dire PB | 10.39 (2.93) | 10.54 (2.87) | 0.349 | |
| Emotional PB | 11.69 (3.48) | 11.53 (3.54) | 0.375 | |
| Public PB | 5.64 (2.37) | 5.38 (1.95) | 0.039 | |
|  |  |  |  | |
| * The results are based on multiple t-tests to illustrate general group differences between classes.  **Abbreviations:** AUDIT = Alcohol Use Disorder Identification Test; DUDIT = Drug Use Disorder Identificaiton Test;  GAD-7 = Generalized Anxiety Disorder-7; PB = Prosocial Behavior, PHQ-9 = Patient Health Questionnaire-9 | | | | |

# Supplementary References

1.	Rozental, A., Sörman, K., Ojala, O., Jangard, S., El Alaoui, S., & Månsson, K. N. T. *et al.* Mental health in individuals with self-reported psychiatric symptoms during the COVID-19 pandemic: Baseline data from a swedish longitudinal cohort study. *Front. Psychiatry* **13**, (2022).

2.	Proust-Lima, C., Philipps, V. & Liquet, B. Estimation of extended mixed models using latent classes and latent processes: The R package lcmm. *J. Stat. Softw.* **78**, (2017).

3.	Nylund, K. L., Asparouhov, T. & Muthén, B. O. Deciding on the number of classes in latent class analysis and growth mixture modeling: A Monte Carlo simulation study. *Structural Equation Modeling* **14**, 535–569 (2007).

4.	Saunders, J. B., Aasland, O. G., Babor, T. F., de la Fuente, J. R., & Grant, M. Development of the Alcohol Use Disorders Identification Test (AUDIT): WHO Collaborative Project on Early Detection of Persons with Harmful Alcohol Consumption-II. *Addiction* vol. 88 (1993).

5.	Berman, A. H., Bergman, H., Palmstierna, T. & Schlyter, F. Evaluation of the Drug Use Disorders Identification Test (DUDIT) in criminal justice and detoxification settings and in a Swedish population sample. *Eur. Addict. Res.* **11**, 22–31 (2005).

6.	Spitzer, R. L., Kroenke, K., Williams, J. B. W. & Löwe, B. A Brief Measure for Assessing Generalized Anxiety Disorder The GAD-7. *Arch Intern Med.* **10**, 1092–1097 (2006).

7.	Kroenke, K., Spitzer, R. L. & Williams, J. B. W. The PHQ-9 Validity of a Brief Depression Severity Measure. *J Gen Intern Med.* 606–613 (2001).

8.	Barkley, R. A. *Barkley Adult ADHD Rating Scale-Iv (BAARS-IV)*. (Guilford Press, 2011).

9.	Foa, E. B., Huppert, J. D., Leiberg, S., Langner, R., Kichic, R., & Hajcak, G. The obsessive-compulsive inventory: Development and validation of a short version. *Psychol. Assess.* **14**, 485–496 (2002).

10.	Blevins, C. A., Weathers, F. W., Davis, M. T., Witte, T. K. & Domino, J. L. The Posttraumatic Stress Disorder Checklist for DSM-5 (PCL-5): Development and Initial Psychometric Evaluation. *J. Trauma. Stress* **28**, 489–498 (2015).

11.	Miller, N. V., Currie, S. R., Hodgins, D. C. & Casey, D. Validation of the problem gambling severity index using confirmatory factor analysis and rasch modelling. *Int. J. Methods Psychiatr. Res.* **22**, 245–255 (2013).

12.	Russell, D. W. UCLA Loneliness Scale (Version 3): Reliability, validity, and factor structure. *J. Pers. Assess.* **66**, 20–40 (1996).

13.	Carlo, G. & Randall, B. A. *The Development of a Measure of Prosocial Behaviors for Late Adolescents*. *Journal of Youth and Adolescence* vol. 31 (2002).

14.	Cohen, J. *Statistical Power Analysis for the Behavioral Sciences*. (routledge, 2013).
